# Engineered ribosomes with tethered subunits for expanding biological function

**DOI:** 10.1038/s41467-019-11427-y

**Published:** 2019-09-02

**Authors:** Erik D. Carlson, Anne E. d’Aquino, Do Soon Kim, Emily M. Fulk, Kim Hoang, Teresa Szal, Alexander S. Mankin, Michael C. Jewett

**Affiliations:** 1 Department of Chemical and Biological Engineering, Northwestern University, 2145 Sheridan Road, Tech E-136, Evanston, IL 60208 USA; 2 Chemistry of Life Processes Institute, Northwestern University, 2170 Campus Drive, Evanston, IL 60208 USA; 3 Center for Synthetic Biology, Northwestern University, 2145 Sheridan Road, Evanston, IL 60208 USA; 4 Interdisciplinary Biological Sciences Graduate Program, Northwestern University, Hogan 2-100, 2205 Tech Drive, Evanston, IL 60208 USA; 5 Center for Pharmaceutical Biotechnology, University of Illinois at Chicago, Chicago, IL 60607 USA; 6 Present Address: Department of Chemical Engineering, Stanford University, Stanford, CA 94305 USA; 7 Present Address: Department of Biology, Johnson and Wales University, Providence, RI 02903 USA

**Keywords:** Translation, Ribosome, Synthetic biology

## Abstract

Ribo-T is a ribosome with covalently tethered subunits where core 16S and 23S ribosomal RNAs form a single chimeric molecule. Ribo-T makes possible a functionally orthogonal ribosome–mRNA system in cells. Unfortunately, use of Ribo-T has been limited because of low activity of its original version. Here, to overcome this limitation, we use an evolutionary approach to select new tether designs that are capable of supporting faster cell growth and increased protein expression. Further, we evolve new orthogonal Ribo-T/mRNA pairs that function in parallel with, but independent of, natural ribosomes and mRNAs, increasing the efficiency of orthogonal protein expression. The Ribo-T with optimized designs is able to synthesize a diverse set of proteins, and can also incorporate multiple non-canonical amino acids into synthesized polypeptides. The enhanced Ribo-T designs should be useful for exploring poorly understood functions of the ribosome and engineering ribosomes with altered catalytic properties.

## Abstract (web summary)

Ribo-T is a tethered ribosome complex capable of orthogonal ribosome-mRNA functionality, but has low activity. Here the authors evolve new tether designs that support faster growth and increased protein expression.

## Introduction

The ribosome is a molecular machine responsible for the polymerization of α-amino acids into proteins^1,2^. In all kingdoms of life, the ribosome is made up of two subunits^3–5^. In bacteria, these correspond to the small (30S) subunit and the large (50S) subunit. The 30S subunit contains the 16S ribosomal RNA (rRNA) and 21 ribosomal proteins (r-proteins), and is involved in translation initiation and decoding the mRNA message^6^. The 50S subunit contains the 5S and 23S rRNAs and 33 r-proteins, and is responsible for accommodation of amino acid substrates, catalysis of peptide bond formation, and protein excretion^7,8^.

The extraordinarily versatile catalytic capacity of the ribosome has driven extensive efforts to harness it for novel functions, such as reprogramming the genetic code^9–13^. For example, the ability to modify the ribosome’s active site to work with substrates beyond those found in nature such as mirror-image (D-α-) and backbone-extended (β- and γ-) amino acids^14,15^, could enable the synthesis of new classes of sequence-defined polymers to meet many goals of biotechnology and medicine^11,16^. Unfortunately, cell viability constraints limit the alterations that can be made to the ribosome.

To bypass this limitation, recent developments have focused on the engineering of specialized ribosome systems. The concept is to create an independent, or orthogonal, translation system within the cell dedicated to production of one or a few target proteins while wild-type ribosomes continue to synthesize genome-encoded proteins to ensure cell viability. Pioneering efforts by Hui and DeBoer^17^, and subsequent improvements by Chin and colleagues^18–21^, first created a specialized small ribosomal subunit. By modifying the Shine-Dalgarno (SD) sequence of an mRNA and the corresponding anti-Shine Dalgarno (ASD) sequence in 16S rRNA, they generated orthogonal 30S subunits capable of primarily translating a specific kind of engineered mRNA, while largely excluding them from translating endogenous cellular mRNAs. These advances enabled the selection of mutant 30S ribosomal subunits capable of re-programming cellular logic^19^ and enabling new decoding properties^20^.

**Fig. 1:**
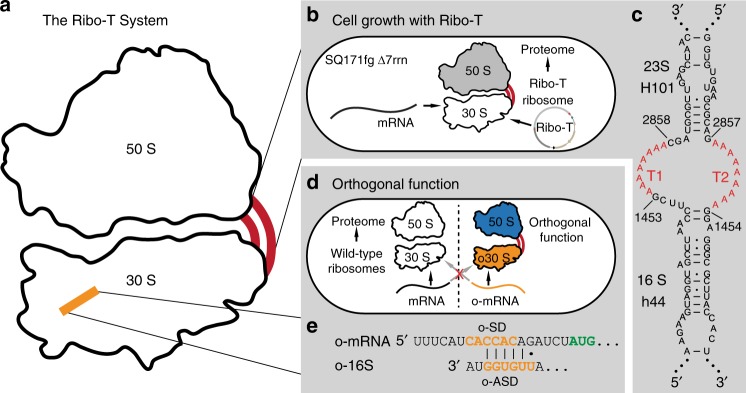
Ribo-T system improvement strategies. **a** Schematic of Ribo-T showing tether (red) and orthogonal ribosome binding site (yellow). **b** The tether is optimized in cells growing exclusively from the Ribo-T plasmid. **c** Previously published Ribo-T tether sequence. **d** Orthogonal function evolved for Ribo-T. **e** Previously published orthogonal mRNA (o-mRNA) Shine-Dalgarno (SD) sequence and orthogonal 16S rRNA anti-SD (o-ASD) sequence shown

Unfortunately, such techniques have been restricted to the small subunit because the large subunits freely exchange between pools of native and orthogonal 30S. This limits the engineering potential of the large subunit, which contains the peptidyl transferase center (PTC) active site and the nascent peptide exit tunnel. We addressed this limitation with a fully orthogonal ribosome (termed Ribo-T), whereby the small and large subunits tethered together via helix h44 of the 16S rRNA and helix H101 of the 23S rRNA (Fig. 1a, c). Not only could this hybrid rRNA be assembled into a functional ribosome in a cell, but Ribo-T could support bacterial growth in the absence of wild-type ribosomes (Fig. 1b). We also used Ribo-T to create the first functionally orthogonal ribosome–mRNA system (Fig. 1d, e), and demonstrated that Ribo-T could be evolved to synthesize protein sequences that the natural ribosome cannot easily translate by selecting otherwise dominantly lethal rRNA mutations in the 50S subunit. This provided the first example of engineering new function in the large subunit of an orthogonal ribosome that was previously inaccessible^22^. Similar results were obtained more recently with an analogously-designed ribosome with conjoined subunits^23,24^. It should be noted that while remaining functionally independent, orthogonal tethered ribosomes still share many components with native translation machinery (e.g., r-proteins, elongation factors and initiation factors)^12^.

Although the functional independence of Ribo-T conceptually enables new opportunities for exploring poorly understood functions of the ribosome, facilitating orthogonal genetic systems, and engineering ribosomes with altered chemical properties, Ribo-T possesses limitations that could hinder its broad applications^25^. For example, cells with only Ribo-T exhibit a slower growth rate than cells with natural wild type ribosomes (doubling time *τ* = 70 ± 2 min as opposed to *τ* = 35 ± 1 min for wild-type), noting that part of this growth rate defect may arise from the circular permutation of the large subunit alone and not the tethering^26^. In addition, the rate of protein synthesis in the Ribo-T cells is ~45% of that of the wild-type^22^ possibly due to slow assembly and the resulting reduced number of functionally-active translating Ribo-T ribosomes^25^. Furthermore, the implemented orthogonal system was simply a modified version of previous works^18,27^, evolved in the context of untethered ribosomes using different plasmid backbones and promoters. Finally, it is not clear if the Ribo-T system is compatible with orthogonal non-canonical amino acid (ncAA) incorporation machinery for applications that could expand the range of genetically encoded chemistry. Taken together, these features of the original Ribo-T system limited some applications.

Here, we address these limitations through the development of an improved Ribo-T design. Specifically, we used evolutionary approaches to select new RNA tethers that connect the 16S and 23S rRNA by sampling an extended pool of tether variants differring in their composition and length. By testing libraries amounting to more than 10^15^ members, we isolated Ribo-T variants with improved properties. Specifically, cells carrying the improved variant, which we term Ribo-T version 2 (Ribo-T v2) has a 24% increase in growth rate (0.75 h^−1^, in SQ171fg strain) as compared to the original Ribo-T (Ribo-T v1; T1: 9A, T2: 8A) and a 12% increase in final OD_600_ at 37 °C as compared to Ribo-T v1 (final Ribo-T v2 OD_600_ = 0.9, in SQ171fg strain). In minimal media, these advantages are even more striking, with Ribo-T v2 possessing a 79% improvement in final OD_600_ at 37 °C relative to Ribo-T v1. We then used directed evolution to improve the orthogonal function of Ribo-T. The optimized orthogonal (o)Ribo-T v2 (mRNA Shine-Dalgarno (SD): 5′-CAACCAC-3′, 16S anti-SD (ASD): 5′-CUGUGG-3′) has a 208% increase in overall expression of the target protein, and possessed a 16% increase in orthogonality (with an orthogonal *cat* reporter) as compared to oRibo-T v1. To demonstrate the utility of the oRibo-T v2, we expressed a diverse set of proteins ranging from small (25 kDA) to large (116 kDa). Lastly, oRibo-T v2 was leveraged to synthesize superfolder green fluorescent protein (sfGFP) possessing multiple, identical ncAAs. Our improvements expand Ribo-T’s applications and make the Ribo-T system better suited for studying and leveraging orthogonal translation in vivo.

## Results

### Tether optimization improves growth of Ribo-T cells

**Fig. 2:**
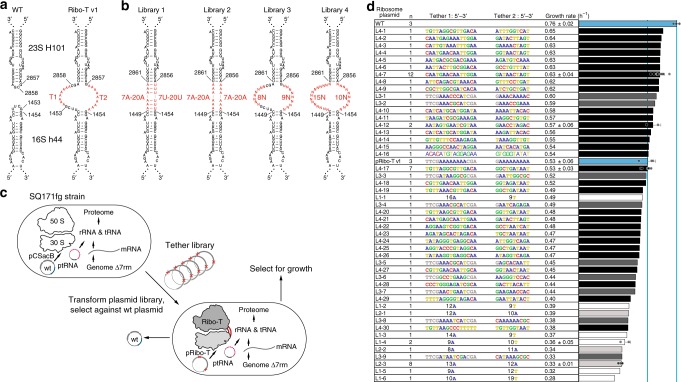
Optimizing tether sequence improves performance. **a** Wild-type 23S rRNA helix 101 and 16S rRNA helix 44 are connected to create Ribo-T with 9A for 5′ tether, T1, and 8A for 3′ tether, T2. **b** Library 1: paired 5′ tether T1 poly A from 7–20 nucleotides, with 3′ tether T2 poly T from 7–20 nucleotides. Library 2: unpaired polyA on both T1 and T2, ranging in 7–20 nucleotides long. Library 3: randomized T1 (8N) and T2 (9N) keeping residues of opened H101 and h44 apex loops. Library 4: randomized apex-to-apex T1 (15N) and T2 (10N) of tether. **c** Selection scheme for improved tethers. Strains lacking genomic copies of rRNA operons (Δ7rrn) are transformed with plasmid-based Ribo-T tether libraries, and the wild-type pCSacB plasmid (wt) is removed. **d** Tether sequences and growth rates of analyzed colonies. Error bars = 1SD of noted independent colonies, n. The top 15 Ribo-T design winners (L4-1 through L4-13) were co-cultured and passaged for 3 days. Between each passage, the bulk culture populations were sequenced and analyzed. Source data for **d** can be found in the Source Data file

We first sought to improve Ribo-T function by optimizing the tether for length and sequence composition (Fig. 2a, b). The original Ribo-T’s (Ribo-T v1) 9-adenine tether T1 connects the 3′ 16S rRNA residue G1453 of helix 44 (h44) to the 5′ 23S rRNA C2858 of helix 101 (H101), and a 8-adenine tether T2 links G2857 of H101 to G1454 of h44 (Fig. 2a). Our initial choice of these oligo(A) tethers for Ribo-T was based on the simplicity of the linker sequence and its presumed resistance to the action of cellular nucleases^22^. We wondered if replacing unpaired linkers with sequences capable of base pairing with each other and forming a double stranded RNA stem would be beneficial for Ribo-T stability and functionality. To test this, we designed four libraries of T1 and T2 tethers at the H101/h44 subunit connection point (Fig. 2b). Libraries 1 and 2 explore tether length in a paired and unpaired format, respectively, without the apex loop remnants present in our original library design^22^. Specifically, library 1 explores tether length with potential base pairing using a 7A-20A T1 tether paired with a 7U-20U T2 tether (for a total library size of 196 members). Library 2 explores a dual poly(A) tether ranging from 7A-20A (196 members). Libraries 3 and 4 explore tether sequence with fixed length of the published pRibo-T tether^22^. Library 3 keeps the apex loop remnants of the original Ribo-T sequence for an 8N/9 N randomized library of 1.7 × 10^10^ members, while library 4 fully randomizes the h44-tether-H101 structure for a 15N/10N randomized library of 1.1 × 10^15^ members, although the entire sequence space was not accessed experimentally because of transformation limitations.

Following library construction (Supplementary Fig. 1), the resulting libraries were individually transformed into the *Escherichia coli* SQ171fg strain^22^, which was evolved from the SQ171 strain^28^ that lacks chromosomal rRNA alleles and survives on the pCSacB plasmid that carries the wt *rrnB* operon and the tRNA67 plasmid that carries missing tRNA genes. The pCSacB plasmid also contains a counter selectable marker *sacB* gene, that confers sensitivity to sucrose. Distinct from the SQ171 strain, the SQ171fg strain contains mutations that were previously shown to improve the growth of the Ribo-T cells^22^. The Ribo-T 23S rRNA in each library contains an A2058G mutation, conferring resistance to erythromycin that facilitates the selection of cells expressing functional Ribo-T. Colonies grew from all libraries in the presence of sucrose (indicating the loss of the pCSacB plasmid) and erythromycin, demonstrating full support of the cellular protein synthesis by tethered Ribo-T expressed from the plasmid (Fig. 2c). Agarose gel electrophoresis of total RNA of a sampling of colonies from each library show the expected dominant Ribo-T size RNA corresponding to the 16S–23S chimera instead of the individual 16S and 23S bands, confirming no significant wild-type ribosome contamination or tether cleavage (Supplementary Fig. 2). Individual colonies (~50–100) were picked from each library (biasing towards bigger colonies), tethers were sequenced, and growth rates were determined (Fig. 2d). While viable clones supported by intact tethered ribosomes were isolated from each library (Supplementary Fig. 2), Library 4 was most successful in yielding clones with improved growth rates compared to pRibo-T v1 (Fig. 2d).

**Fig. 3:**
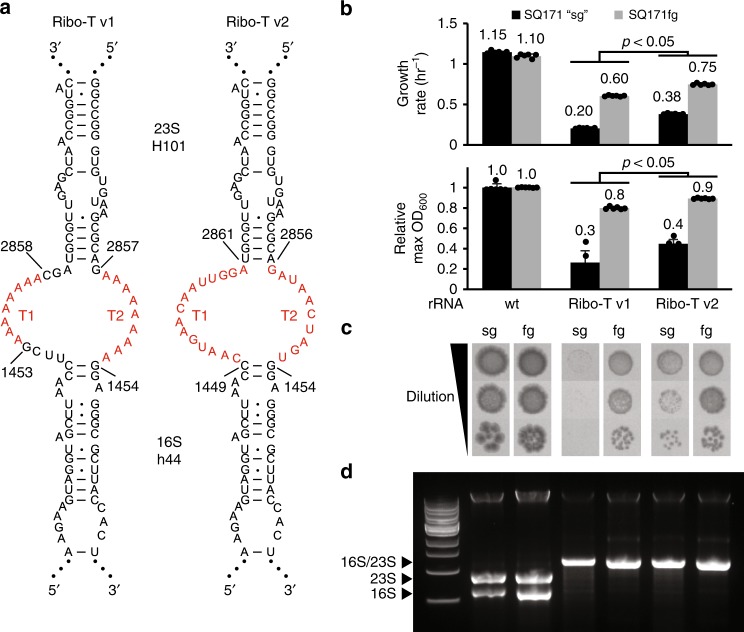
Optimizing tether sequence improves performance. **a** Ribo-T v1: previously published tether sequence. Ribo-T v2: fastest growing and most frequent selected tether sequence. **b** Growth rate and max OD_600_ of SQ171 slow growing (sg) and SQ171 and fast growing (fg) cells growing with pAM552 (wild-type *rrnb* operon), pRibo-T v1 and pRibo-T v2 (*n* = 6; paired *t*-test [two-sided], *p* < 0.05). Error bars = 1SD. **c** Spot plated SQ171 and SQ171fg cells growing with pAM552, pRibo-T v1 and pRibo-T v2 imaged after 48 h at 37 °C. **d** Total RNA extraction from SQ171 and SQ171fg cells growing with pAM552, pRibo-T v1 and pRibo-T v2. Source data for **b**–**d** can be found in the Source Data file

We next carried out additional evolutionary experiments to let the cells with the top 15 most improved tether sequences that emerged from this selection compete in liquid culture. Specifically, the top 15 strains (Fig. 2d, L4–1 through L4-13) were individually grown in separate liquid cultures, combined at equal OD_600_ in co-culture, in triplicate, and passaged for three days. Between each passage, both the bulk populations and individual resultant colonies from plated cultures were sequenced and analyzed (Supplementary Fig. 3). After 3 passaging days, all three cultures converged to sequence L4-7, which we term Ribo-T v2 (Fig. 3a).

In both liquid culture growth (Fig. 3b) and plate growth assays (Fig. 3c), cells supported exclusively by pRibo-T v2 outperform pRibo-T v1 in both SQ171 and SQ171fg strains. Specifically, in the SQ171fg strain, the pRibo-T v2 plasmid improves growth rate by 24% and the maximum OD_600_ in LB media by 12% as compared to the pRibo-T plasmid (*n* = 6, paired t-test [two-sided], *p* < 0.05). The benefits are more pronounced in the original SQ171 strain, where growth rate improves by 86%, and max OD_600_ by 70% as compared to pRibo-T (*n* = 6, paired t-test [two-sided], *p* < 0.05). The growth curves also highlight a significantly reduced lag time in cell growth for Ribo-T v2 cells versus Ribo-T v1 cells in both SQ171 and SQ171fg strains (Supplementary Fig. 4). Agarose gel electrophoresis of total RNA extracted from cells supported by pRibo-T v2 plasmids show the expected 16S–23S sized RNA, and the loss of individual 16S and 23S rRNA bands (Fig. 3d).

We next tested if the Ribo-T v2 growth improvement properties were robust, by comparing growth relative to Ribo-T v1 in different strains (i.e., SQ171, SQ171fg, POP2136), at various growth temperatures (30, 37, and 42 °C) and different media (Supplementary Fig. 5). We observed appreciable improvements in each case. The advantage of Ribo-T v2 was especially pronounced at 30 °C in M9-casamino acids (M9CA) minimal media with a 78% and 69% improvement, respectively, in final max OD and average doubling time over Ribo-T v1 (Supplementary Fig. 5e, f). Since the Ribo-T v2 design showed superior growth characteristics, remained uncleaved, and outperformed other tether sequences in a liquid culture competition, this construct was selected for future experiments.

While we do not have a simple explanation for why the newly selected tethers improve the growth rate of Ribo-T v2 cells relative to Ribo-T v1, it may be attributed to the possible partial pairing of the new tethers. Specifically, chemical probing and modeling of the secondary structure^29^ suggest that a segment of the tethers may form a base-paired duplex (Supplementary Fig. 6). Conceivably, the structure of the improved tethers may either facilitate the Ribo-T v2 assembly, which as we know is one of the main limiting properties of the original Ribo-T design^25^ or may better facilitate the relative movement of the tethered subunits during initiation, elongation of termination steps of translation.

### Improvement of Ribo-T orthogonal function

After selecting optimized tethers, we sought to improve the orthogonality of the tethered ribosome system. Orthogonal function of Ribo-T is achieved by altering the mRNA SD sequence and the corresponding ASD sequence of the 16S rRNA. In this way, a specialized pool of orthogonal Ribo-T (oRibo-T) is created that exclusively translates the cognate mRNA and in principle, should be functionally isolated from the pool of wild-type mRNA and ribosomes. Our oRibo-T system^22^ utilized a modified version of a previously developed orthogonal 30S subunit system^18^, not one developed in the Ribo-T context. We hypothesized that because initiation with Ribo-T is limiting^22,25^, optimizing the SD/ASD pairing could improve orthogonal system functionality.

**Fig. 4:**
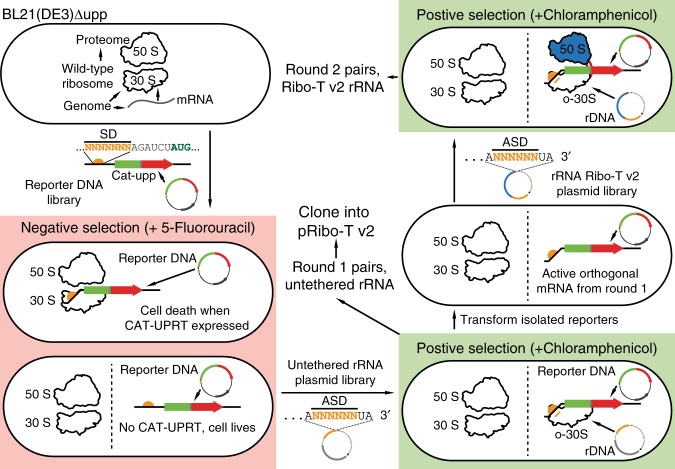
Improving orthogonal pairs. Selection scheme to optimize orthogonal Shine-Dalgarno (SD) and anti-Shine-Dalgarno (ASD) pairs in untethered and tethered context

The goal of this effort was to improve orthogonal protein expression by oRibo-T v2, while minimizing cross-talk of the orthogonal mRNA with wild-type ribosomes. To this end, we used a robust directed evolution approach^18^ to select highly functional and orthogonal Ribo-T v2/mRNA pairs (Fig. 4). Specifically, a fusion of the *cat* and *upp* genes (Supplementary Fig. 7a) enables both a positive and a negative selection from a single gene product: chloramphenicol acetyltransferase encoded in the *cat* gene confers resistance to chloramphenicol (Cm), whereas the fused *upp* gene codes for uracil phosphoribosyltranferase causing cell death in the presence of 5-fluorouracil (5-FU) (Supplementary Fig. 8).

For the negative selection step, the wild-type SD sequence (5′-AAGGAGG-3′) for the *cat-upp* gene on plasmid plpp5-catupp-p15A (Fig. 4, Supplementary Fig. 7a) was entirely randomized. We then transformed the plasmid library into BL21(DE3)Δupp cells and plated on M9 minimal media agar plates supplemented with 10 µg ml^−1^ 5-FU. Surviving cells produce mRNA that is not efficiently translated by endogenous ribosomes (desired outcome), or have non-functional plasmids. In the initial attempts of the subsequent positive selection, we had difficulty selecting robust orthogonal SD/ASD pairs from this o-SD mRNA pool with a randomized ASD-Ribo-T library directly. Therefore, we performed a first round of positive selection using untethered ribosomes with the small subunit carrying randomized ASD in order to limit the o-mRNA sequence space to just orthogonal and sufficiently active o-mRNA sequences. Specifically, the 16S rRNA ASD sequence of plasmid-based untethered ribosomes (Supplementary Fig. 7b) was randomized, the plasmids were transformed into the surviving cells from our negative selection, and then plated on LB-agar plates in the presence of 100 µg ml^−1^ Cm. Surviving colonies were picked, and plasmids were isolated and sequenced (Round 1, Supplementary Fig. 9b).

To identify top performing o-mRNAs, we evaluated the round 1 selected SD/ASD pairs for overall reporter expression levels and assessed the extent of cross-talk with wild-type. This initial characterization of orthogonal SD/ASD pair activity was performed using a Cm-resistance assay and the *cat-upp* reporter plasmids. To test overall activity, each set of cognate o-mRNA and o-16S rRNA plasmids was added to the same cells and resistance to Cm assessed. Additionally, to measure orthogonality of the corresponding mRNAs with the wild-type ribosome pool (i.e., how much cross-talk exists between wild-type ribosomes and our selected orthogonal mRNAs), each orthogonal mRNA construct was independently co-transformed into fresh BL21(DE3)Δupp cells with plasmid coding for wild-type ribosomes (pAM552, Supplementary Fig. 7b). Round 1 strains were plated on a range of Cm concentrations (0, 0.5, 1, 2.5, 5, 10, 20, 40, 60, 80, 100, 200, 300, 400, and 500 μg ml^−1^), and maximum growth concentrations noted (Supplementary Fig. 9a). Evolved pairs had increased cognate pair activity (black bars) well above the background expression of the o-mRNA by wild-type ribosomes (white bars). Furthermore, orthogonal pair activity was significantly increased over the previous orthogonal system^22^ (pAM552o/A, Supplementary Fig. 9).

**Fig. 5:**
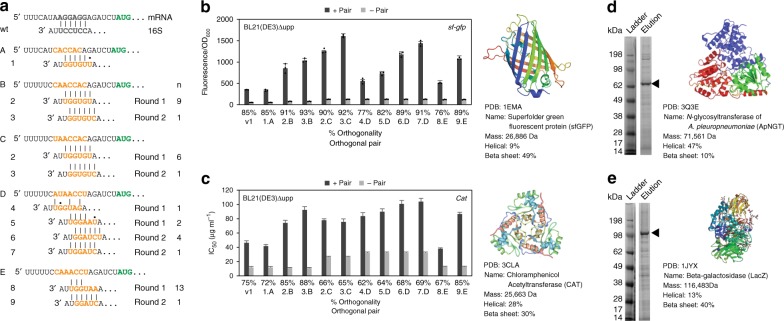
Selected orthogonal pair sequences and function in Ribo-T v2. **a** Top evolved orthogonal mRNA and 16S with predicted pairing. Selection round is noted by round 1 or round 2 to the right of each pair. n denotes number of isolated members with that sequence from the selection. **b**–**e** Orthogonal pair notation: Original orthogonal Ribo-T system denoted by v1, and x.y where x is o16S number and y is o-mRNA letter (pORTx.y plasmid name format). **b** Orthogonal expression of super folder green fluorescent protein (*sf-gfp*) in BL21(DE3)Δupp.+ pair: both o-rRNA and o-mRNA expressed, − pair: just o-mRNA expressed without cognate o-rRNA. Percent orthogonality is shown below column labels. A higher percentage value is desired, indicating a lower background expression of o-mRNA as compared to the expression with the cognate orthogonal rRNA. Error bars = 1SD of *n* = 3 independent experiments. The protein’s structure and details are listed to the right of the graph. **c** Orthogonal expression of Cm acetyltransferase (*cat*) in BL21(DE3)Δupp. Error bars = 1 standard error in IC_50_ curve fitting. The protein’s structure and details are listed to the right of the graph. **d** Orthogonal expression of *N*-glycosyltransferase of *A. pleuropneumoniae* (*ApNGT*) in BL21(DE3). The protein’s structure and details are listed to the right of the graph. **e** Orthogonal expression of Beta-galactosidase (LacZ) in BL21(DE3). The protein’s structure and details are listed to the right of the graph. Source data for **b**, **c** can be found in the Source Data file

We used a set of 14 best-performing orthogonal mRNAs for a second round of positive selection with a library of Ribo-T v2 with the ASD sequence randomized. First, the active and orthogonal mRNA (o-mRNA B-P, Supplementary Fig. 9b) were isolated, pooled and transformed into the BL21(DE3)Δupp strain. Then, the ASD sequence on pRibo-T v2 plasmid was randomized, transformed into BL21(DE3)Δupp containing the top performing orthogonal mRNAs, and plated on LB-agar plates supplemented with 100 μg ml^−1^ Cm (Fig. 4). Surviving colonies were picked, and plasmids were isolated and sequenced. Top performing pairs, aligned using the ribosome binding site (RBS) calculator^30,31^, are shown in Fig. 5a. The alignments show that while the selected orthogonal SD/ASD pairs are different from wild-type sequences, they have high complementarity between themselves. Our orthogonal Ribo-T constructs with improved v2 tethers are named pORTx.y, where x is a number indicating the orthogonal ASD sequence (1–9), and y is a letter indicating the corresponding cognate SD sequence (A–E). Corresponding rRNA plasmids with untethered ribosomes are named pOx.y.

### Evaluation of evolved orthogonal pairs

With improved orthogonal Ribo-T v2/mRNA pairs in hand, we assessed performance with two key metrics: (i) the overall activity and (ii) the orthogonality to wild-type ribosomes. Pair activity and orthogonality were measured with two protein expression assays: fluorescent protein expression and antibiotic resistance. These two assays were chosen to validate and demonstrate that these orthogonal Ribo-T v2 exhibit comparable relative orthogonal expression regardless of the protein they express. Importantly, a metric for quantifying orthogonality is critical, because it segregates the activity of oRibo-T v2 from that of wild-type ribosomes, and normalizes orthogonality across the two different assays. Percent orthogonality is calculated as:

1 $$\% \,{\mathrm{orthogonality}} = \frac{{{\mathrm{A}}_{{\mathrm{pair}}} - {\mathrm{A}}_{{\mathrm{mRNA}}}}}{{{\mathrm{A}}_{{\mathrm{pair}}}}} \times 100$$

Where A_pair_ is the activity of the orthogonal pair (sfGFP fluorescence divided by OD_600_ for the fluorescent protein expression assay, or half maximal inhibitory concentration (IC_50_) for the CAT assay), and A_mRNA_ is the activity of just the orthogonal mRNA expressed without the cognate orthogonal ribosome (i.e., the crosstalk with wild-type ribosomes). The extent of orthogonality (%) is shown below each pair in the activity plots in Fig. 5. With this metric, a higher percentage value indicates a lower background expression of o-mRNA in the absence of cognate oRibo-T v2 as compared to the expression when the cognate oRibo-T v2 is present.

For evaluation of selected orthogonal pairs, SD variants were cloned into vectors containing the *sf-gfp* and *cat* genes, respectively. ASD variants were freshly cloned into the pRibo-T v2 plasmid. Plasmid pairs were transformed into a naïve BL21(DE3)Δupp strain for testing. Expression of sfGFP was measured as final fluorescence normalized by the final OD_600_ reading (Fig. 5b) and activity of CAT was evaluated as IC_50_ (Fig. 5c). Of note, pair activity is improved in both sfGFP and CAT assays over the original published oRibo-T system^22^ (noted as v1), as well as the published v1 orthogonal pair cloned with the optimized v2 tether sequences (noted as 1.A). We observed that some pairs achieved high sfGFP expression (*e.g*., pORT3.C, Fig. 5b), other pairs conferred particularly strong resistance to Cm (e.g., pORT7.D, Fig. 5c), some pairs achieved high orthogonality (e.g., pORT3.B, Fig. 5b, c), some pairs had moderate activity but poor orthogonality (e.g., pORT4.D, pORT8.E, Fig. 5b, c), and some pairs achieved a balance of high activity and orthogonality e.g., pORT2.B, pORT3.B, Fig. 5b, c). When considering both assays, and metrics of pair activity and orthogonality, we selected o-mRNA B (oSD: 5′-CAACCAC) paired with o-ASD #2 (5′-UGUGGU) (selected in Round 1 in untethered context), and o-ASD #3 (5′-CUGUGG) (selected in Round 2 in v2 tether context).

To directly compare performance of the newly selected orthogonal pairs against our original orthogonal pair^22^, we cloned the previous o-ASD sequence into the Ribo-T v2 plasmid to generate pORT1, and the cognate orthogonal SD sequence into the *sf-gfp* and *cat* reporter plasmids to generate plpp5.A.gfp and plpp5.A.cat (Fig. 5a). For plasmids pORT2 and pORT3 paired with orthogonal GFP reporter B (plpp5.B.gfp), we observed activity increases of 154% and 208%, respectively, compared to pORT1. Percent orthogonality also increased by 6% and 8% (*n* = 6, paired *t*-test [two-sided], *p* < 0.05), respectively (Fig. 5b). For plasmids pORT2 and pORT3 paired with orthogonal *cat* reporter B (plpp5.B.cat), pair activity increased 77% and 121% over pORT1, respectively. Percent orthogonality increased 13% (for 2.B) and 16% (for 3.B) over pORT1, respectively (Fig. 5c). While the orthogonal GFP reporter C (plpp5.C.gfp) had higher functionality than the orthogonal GFP reporter B (plpp5.B.gfp) with pORT2 and pORT3, its orthogonality was lower than that of the reporter B (Fig. 5b, c). The new mRNA/oRibo-T pairs (o-mRNA B: 5′-CAACCAC; o-ASD #3: 5′-CUGUGG) are poised to expand the versatility of the fully orthogonal ribosome–mRNA system.

### Orthogonal pair activity in other E. coli strains

To test system versatility in a wide range of strains, top performing plasmid pairs for the sfGFP reporter set were next transformed into BL21 Star (DE3) (Invitrogen) and a variant of the fully recoded C321.∆A strain^32,33^, MCJ1217. These strains provide benefits for ncAA incorporation using amber suppression and we recently showed that C321.∆A could be coupled with extensively engineered synthetases for multi-site incorporation of up to 30 ncAAs into a single biopolymer in vivo^34^ and developed for cell-free protein synthesis applications as well^33,35–37^. Following transformation, we evaluated the ability of our top performing oRibo-T v2/o-mRNA pairs to express sfGFP (Supplementary Fig. 10a). General trends observed in the BL21(DE3)Δupp strain hold for these additional strains: pORT2.B, pORT3.B, pORT2.C and pORT3.C sets perform better than the original pair (>200% of pORT1 expression under similar conditions), with maintained high orthogonality. The best-performing orthogonal pairs similarly benefitted specialized 30S subunits in a non-tethered context (Supplementary Fig. 11).

### Synergistic effect of evolved tethers and orthogonal pairs

We next set out to study the effects of improved tethers and orthogonal pairs on the oRibo-T system performance. To do this, select orthogonal ASD sequences were cloned into both our improved oRibo-T v2 plasmid as well as our original published oRibo-T v1 (with tether sequences 9A/8A)^22^. Using both our orthogonal sfGFP and CAT assays, we measured the activity (fluorescence for sfGFP, and IC_50_ for CAT) of our orthogonal pairs in the context of either Ribo-T v1 or v2 tethers. In our sfGFP assay, we observed improvements in activity and orthogonality for Ribo-T v2 when combined with every orthogonal pair. Specifically, v2 tethers and improved orthogonal pairs worked synergistically to improve orthogonal function over the v1 tethers by up to 55% (Supplementary Fig. 12a). The CAT assay did not show significant difference between v1 and v2 tethers (Supplementary Fig. 12b), presumably because of the less sensitive assay range compared to the sfGFP fluorescence assay.

To further demonstrate the utility of the oRibo-T v2 system, we expressed additional recombinant proteins aiming to represent a diverse range of protein sizes, structures, and functions. Specifically, we cloned *E. coli β*-galactosidase (LacZ) and *N*-glycosyltransferase of *A. pleuropneumoniae* (ApNGT) into our in vivo orthogonal reporter construct (plpp5.B). We then purified the encoded proteins, and compared their expression across oRibo-T v2 and oRibo-T v1 (Fig. 5d, e and Supplementary Fig. 10b–d). Importantly, cells carrying oRibo-T v2 had a 37% higher expression of LacZ and a 22% higher expression of ApNGT over oRibo-T v1 (*n* = 3, paired *t*-test [two-sided], *p* < 0.05). These results demonstrate Ribo-T v2’s utility in producing a variety of proteins of various sizes (25–116kDa), structural compositions (9–47% alpha helical and 10–49% beta sheets), and functions (fluorescence, antibiotic resistance, hydrolysis, and glycosylation).

### Incorporation of non-canonical amino acids by Ribo-T

Engineering the translation apparatus is a key emerging opportunity in synthetic biology^38–40^. One of the central reasons to develop an orthogonal Ribo-T system is the possibility of selecting otherwise dominantly lethal rRNA mutations in the peptidyl transferase center that facilitate the translation of new abiological polymers made with the use of an expanded genetic code^9,39^. Such efforts require that the Ribo-T platform is compatible with orthogonal ncAA incorporation machinery and, up to now, compatibility has yet to be shown in the Ribo-T system, and multiple ncAA incorporations with a tethered o-ribosome has yet to be achieved.

We therefore tested whether oRiboT is compatible with multiple site-specific ncAA incorporation into proteins. Specifically, we assessed the ability of orthogonal Ribo-T v2 (pORT3) to site-specifically incorporate *p*-azido-l-phenylalanine (pAzF) into sfGFP, using a previously reported orthogonal transfer RNA (tRNA) and aminoacyl-tRNA synthetase (aaRS) pair from *Methanocaldococcus jannaschii*^41^ (henceforth referred to as pAzFRS). Importantly, the idea was not to engineer oRibo-T to be better than a natural ribosome at incorporating pAzF, which is known to be incorporated efficiently, but rather to show that oRibo-T and the pAzF orthogonal translation system were able to cooperate in producing protein(s) with multiple ncAAs.

To minimize plasmid requirements for ncAA incorporation, we first combined the oRibo-T v2 rRNA and the reporter gene on one plasmid. Since relative directional orientation of the two expression cassettes from a single plasmid can have a significant impact on system performance^42–44^, we built and tested combined rRNA/mRNA plasmids in both the forward and reverse directions (Supplementary Fig. 13a). While pORT3.B.gfp forward and reverse constructs had similar overall expression, the growth characteristics of the reverse orientation was significantly better than the forward orientation (Supplementary Fig. 13b, graph inset), and so this orientation was selected for future experiments.

**Fig. 6:**
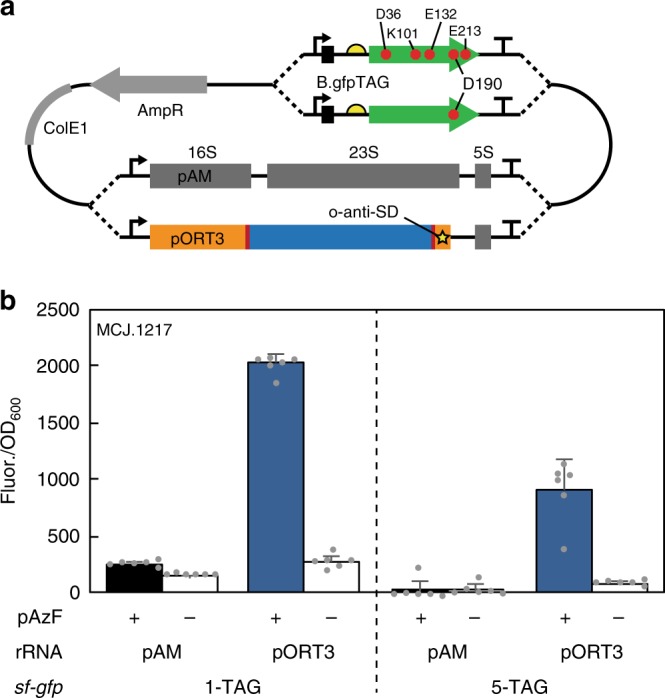
Incorporation of ncAA p-azido-l-phenylalanine (pAzF) by orthogonal Ribo-T. **a** Combined rRNA and *sf-gfp* plasmid with *sf-gfp* gene is replaced with a 1TAG or 5TAG version to create pORT3B.gfp1TAG and pORT3B.gfp5TAG (orthogonal Ribo-T with ASD sequence 3 and orthogonal sfGFP message B containing 1 TAG or 5 TAG, respectively). Wild-type *rrnb* operon was cloned as a negative control for background orthogonal expression (pAM.B.gfp1TAG and pAM.B.gfp5TAG). **b** Expression of *sf-gfp* with 1TAG or 5TAG in C321.ΔA derived strain MCJ.1217 (C321.ΔA.mutS^+^.Δλred.Δupp), in the presence of (+) or absence of (−) pAzF. Error bars = 1SD of *n* = 6 independent experiments. Source data for **b** can be found in the Source Data file

We then tested ncAA incorporation. The genomically-recoded organism derived from C321.∆A (MCJ.1217) lacking UAG stop codons was co-transformed with our combined reporter gene and an orthogonal translation system plasmid containing an aaRS:tRNA pair previously engineered for incorporation of pAzF^41^. We quantitatively assessed the incorporation of pAzF into sfGFP variants with 1 or 5 TAG codons at amino acid positions D190 (1 TAG) or D36, K101, E132, D190, and E213 (5 TAG) (Fig. 6a, Supplementary Fig. 13c). Cells containing plasmids encoding for orthogonal Ribo-T with ASD sequence 3 and orthogonal sfGFP message B containing 1 TAG (pORT3B.gfp1TAG) or 5 TAG (pORT3B.gfp5TAG) were grown in LB media supplemented with pAzF. Upon analyzing fluorescence, we found oRibo-T v2 to be successful in translating the *sf-gfp* gene containing not only one TAG but even five internal TAG codons with expression levels >six-fold and >10-fold above background, respectively (Fig. 6b). The expression levels are statistically significant (paired t-test [two-sided], *p* < 0.05) and in line with previously reported values in the literature for this system configuration^45^. Similar expression was observed with the untethered orthogonal ribosome system with plasmids pO2B.gfp1TAG and pO2B.gfp5TAG (Supplementary Fig. 13d). Our results highlight the effective utility of our combined plasmid design for incorporation of ncAAs. Furthermore, our work demonstrates a key proof-of-concept result that confirms compatibility and utility of a Ribo-T v2-based orthogonal system with widely used and standardized orthogonal translation components^33,45,46^.

## Discussion

Here, we present improvements to the original Ribo-T platform. This second-generation design was developed using tether libraries varying in both the length and composition of the tether sequence. We identified several sequences at the h44/H101 junction capable of supporting robust cell growth with the construct carrying T1 (CAATGAACAATTGGA) and T2 (GATAACTAGT) being the winning variant. The new Ribo-T v2 system exhibits up to an 86% improvement in growth rate and 70% improvement in maximum OD_600_ (in SQ171 strain), as compared to the original Ribo-T v1. The improvement in tether design was insufficient to bring the growth rate of the Ribo-T v2 cells to that of wild type cells. We believe this reflects a fundamental limitation of this Ribo-T architecture, which is based on insertion of a circularly permutated 23S rRNA into a 16S rRNA helix at H101 and h44. The unusual structure and transcription order of the rRNA segments in Ribo-T causes notable assembly defects^25^. We are not sure whether it is the circular permutation of the large subunit rRNA or disruption of the continuity of the small subunit rRNA that are the primary cause of the assembly problems. However, in spite of assembly limitations, Ribo-T v2 has marked improvements over the original Ribo-T variant. Furthermore, after the selection of enhanced orthogonal Ribo-T v2/mRNA pairs, orthogonal Ribo-T v2 (pORT3) exhibits a ~200% increase in activity for sfGFP expression and also improved orthogonality compared to our original orthogonal system^22^.

The improvements presented here to the Ribo-T platform enhance the usefulness of the system for biochemical assays (e.g., faster growth for RNA extractions, and higher density cultures for increased preparation of Ribo-T v2 variants for in vitro utilization), and applications. Specifically, these improvements allowed us to demonstrate the usefulness of the orthogonal mRNA-Ribo-T v2 system for two different applications. We demonstrated that orthogonal Ribo-T v2 is capable of synthesizing a range of diverse proteins of different sizes, structures, and functions with enhanced efficiency over Ribo-T v1. Second, as a proof of concept, we demonstrated that oRibo-T can be leveraged for the site-specific incorporation of multiple ncAAs into proteins. We showed successful Ribo-T mediated incorporation of up to five pAzF residues with >10-fold expression above background.

Looking forward, the new Ribo-T v2 is expected to become a versatile tool for many biotechnology, engineering, and basic science applications. These applications and opportunities have sparked enthusiasm, resulting in parallel work featuring a conceptually similar design of an orthogonal stapled ribosomes^23,24^. Although the stapled ribosomes leveraged our same circular permutation and helix connections found in the Ribo-T design (H101 and h44)^22,47^, recently reported improvements to the initial stapled system yielded a strain carrying tens of mutations within the evolved strain^24^, which leaves some uncertainty about portability of that system. Our Ribo-T v2 construct was originally developed in a widely used strain^28^, and is portable and functional in several other strains without extensive strain modifications. These attributes make our orthogonal Ribo-T v2 system robust for a variety of applications and studies. This includes modifying the catalytic capacity of the ribosome for improved incorporation of ncAAs such as backbone-extended monomers (e.g., β*-*, D-, or γ- amino acids)^48,49^ into polypeptides and biopolymers, probing single and multi-mutations in highly conserved rRNA nucleotides, translation of problematic protein sequences, and the creation of an orthogonal central dogma, which may insulate genetic programs from host regulation and allow expansion of the roles of these processes within the cell^12^.

## Methods

### Construction of the tether libraries

Plasmid construction and DNA manipulations were performed following standard molecular biology techniques. The libraries of tether sequences were introduced into the wild-type pRibo-T plasmid by inverse PCR amplification with Phusion polymerase (NEB) with primers listed in Supplementary Table 1. All primers were synthesized by Integrated DNA Technologies. Amplification was followed by re-circularization with the Gibson assembly reaction^50^ (Supplementary Fig. 1). Specifically, Ribo-T backbone plasmid was prepared by PCR amplification with primers 5′-GGAGGGCGCTTACCACTTTG and 5′-GGTTAAGCTACCTACTTCTTTTG using pRibo-T^22^ as template. Using Phusion polymerase, PCR was performed at 98 °C initial denaturing for 3 min, (98 °C 30 sec, 55 °C 30 sec, 72 °C 70 sec)x25, and 72 °C final extension for 10 min. This amplifies the pRibo-T vector, excluding the tethers and 23S region of the plasmid.

To generate the tether libraries (Fig. 2b), primer pools were first prepared from primers listed in Supplementary Table 1. For library 1, equimolar amounts of primers T1-A7-f through T1-A20-f were mixed to create the forward primer pool, and equimolar amounts of primers T1-T7-r through T1-T20-r were mixed to create the reverse primer pool. For library 2, equimolar amounts of primers T1-A7-f through T1-A20-f were mixed to create the forward primer pool, and equimolar amounts of primers T1-A7-r through T1-A20-r were mixed to create the reverse primer pool. Library 3 is generated using primers T1-8N-f and T2-9N-r. Library 4 is generated using primers T1-15N-f and T2-10N-r. In four separate PCRs under the same reaction conditions just described, respective library primers were used with template pRibo-T to generate PCR products of tether libraries flanking CP23S rRNA (Supplementary Fig. 1). Following gel extraction of the Ribo-T backbone and 4 tether libraries from 0.7% agarose gels with E.Z.N.A. gel extraction kit (Omega), 50 ng of Ribo-T backbone was re-circularlized in four separate Gibson assembly reactions with three-fold molar excess of respective libraries. Two microliters of each library was transformed into POP2136 cells (F^−^*glnV44 hsdR17 endA1 thi-1 aroB mal*^−^*cI857* λ *PR* Tet^R^) via electroporation and incubated at 30 °C to repress expression of the p_L_ promoter with POP2136 constitutively expressed cI repressor. In all, 40–80 colonies were selected from each library plate and library diversity was verified by DNA sequencing (Northwestern Sequencing Core). For each library, transformations and plating was scaled until total number of colonies exceeded 3x the theoretical library sizes. Plates were then washed and miniprepped with the E.Z.N.A miniprep kit (Omega) to prepare the four plasmid libraries.

### Replacement of the wild-type ribosome by Ribo-T v2

SQ171 and SQ171fg cells harboring the pCSacB plasmid were transformed with the Ribo-T v2.0 library preparations (Supplementary Fig. 1). In brief, 20 ng of plasmid was added to 50 μL of electrocompetent cells. Cells were resuspended in 800 μL of SOC and incubated for 1 h at 37 °C with shaking. A 250 μL aliquot of recovering cells was transferred to 1.85 ml of SOC supplemented with 50 μg ml^−1^ of carbenicillin and 0.25% sucrose (final concentrations) and grown overnight at 37 °C with shaking. Cells were spun down and plated on LB agar plates containing 50 μg ml^−1^ carbenicillin, 5% sucrose and 1 mg ml^−1^ erythromycin.

### Selecting mutants and analyzing tethers

Colonies that appeared after 24–48 h incubation of the plates at 37 °C were inoculated in a Costar flat bottom 96-well plate containing 100 μL of LB supplemented with 50 μg ml^−1^ carbenicillin and 1 mg ml^−1^ erythromycin. Growth rates were monitored at 37 °C in a BioTek microplate reader. Absorbance at 600 nm was read every 10 min (continuous linear shaking with a 2-mm amplitude). Doubling times were calculated from the growth curve readings during logarithmic growth as determined by regression.

The fastest growing tether mutants were inoculated in 2 ml LB supplemented with 50 μg ml^−1^ carbenicillin, 5% sucrose and 1 mg ml^−1^ erythromycin and grown for 24–48 h. Plasmids were isolated from clones and tethers were sequenced (Northwestern Sequencing Core). Tether composition and library diversity were analyzed by sequencing with primers 5′- GCTGTCGTCAGCTCGTGTTG-3′ for T1 site and 5′-CTGGAGAACTGAGGGG-3′ for T2 site.

### Liquid culture competition assay

The top 15 Ribo-T v2 tether winners identified in the initial library screen were transformed individually into SQ171fg cells. Each were grown individually in separate liquid cultures. The cultures were grown overnight at 37 °C, with shaking, in LB supplemented with 50 μg ml^−1^ carbenicillin and 1 mg ml^−1^ erythromycin. After ~18 h, the OD_600_ of each culture was measured. Equal OD_600_ units of each culture were combined into a co-culture, in triplicate, and passaged for 3 days. Between each passage, both the bulk populations and individual resultant colonies from plated culture were sequenced via sanger sequencing and analyzed.

### Total RNA analysis of tethered Ribo-T v2

Successful replacement of the wild type of pCSacB plasmid with the pRibo-T plasmids carrying Ribo-T v2 was confirmed via total RNA extraction. Total RNA was extracted from these clones using RNeasy Mini Kit (Qiagen) and analyzed by agarose gel electrophoresis (Supplementary Fig. 2).

### Selection of new orthogonal pairs

Before selection could be carried out for a highly orthogonal and active 16S/mRNA pair, the BL21(DE3)Δupp strain was prepared by deleting the genomic copy of *upp* from the BL21(DE3) strain using Datsenko-Wanner recombination^51^ and replacement with a kanamycin resistance (KanR) cassette. The deletion cassette was PCR amplified from pKD4 plasmid^51^ with primers 5′-AATCCGTCGATTTTTTTTGTGGCTGCCCCTCAAAGGAGAAAGAGTTGTGTAGGCTGGAGCTGCTTC and 5′-AAAAAAAAGCCGACTCTTAAAGTCGGCTTTAATTATTTTTATTCTGTCCATATGAATATCCTCCTTAG, with Phusion polymerase (NEB) and 98 °C initial denaturing for 3 min, (98 °C 30 s, 55 °C 30 s, 72 °C 2 min) × 25, and 72 °C final extension for 10 min. Plasmid pCP20 was transformed into a kanamycin-resistant colony to remove the KanR cassette by the incorporated flippase sites^51^. Transformed cells were plated on LB agar supplemented with 50 μg ml^−1^ carbenicillin and grown overnight at 30 °C. Colonies were picked, plated on LB agar plates, and grown overnight at 42 °C to select for loss of pCP20 plasmid. Colonies were checked for kanamycin sensitivity, and deletion was confirmed by sequencing of PCR product from colony PCR using primers 5′-TGCCAGGGTAAAGGTTAG and 5′-GACGGTTGCACCAAAC, and Multiplex PCR mix (Qiagen), flanking the deletion site.

For plasmid compatibility with the rRNA pAM552 plasmid backbone, the origin of replication on pLpp5oGFP^22^ was first switched from pMB1 to p15A. Plasmid origin of replication p15A was synthesized by IDT as a gBlock (Supplementary Table 1), and amplified using primers 5′-GATGGCCTTTTTGCGTTTC and 5′-CTGAGAGTGCACCATACAG with Phusion polymerase (NEB) and 98 °C initial denaturing for 3 min, (98 °C 30 sec, 55 °C 30 s, 72 °C 30 s) × 25 cycles, and 72 °C final extension for 10 min. Plasmid pT7wtK^22^ was amplified with primers 5′- GGATCTGTATGGTGCACTC and 5′- TGTAGAAACGCAAAAAGGCCATC with 98 °C initial denaturing for 3 min, (98 °C 30 sec, 55 °C 30 sec, 72 °C 2 min) × 25 cycles, and 72 °C final extension for 10 min. Following digestion with DpnI (NEB), correct sized DNA was gel extracted from a 0.7% agarose gel with E.Z.N.A. gel purification kit (Omega). Using Gibson assembly^50^, 50 ng of backbone was recircularized with three-fold molar excess of p15A insert and transformed into DH5α electrocompetent cells, plated on LB agar plates supplemented with 30 µg ml^−1^ kanamycin and isolated for sequence confirmation.

Next, *cat-upp* gene was prepared from pRepCM3 plasmid^52^, containing an internal TAG codon for amber suppression. The TAG codon was mutated back to CAA with inverse PCR using primers 5′- CACCCTTGTTACACCGTTTTCCATGAGCAAACTGAAACGTTTTCATCGCTC and 5′- CTCATGGAAAACGGTGTAAC, pRepCM3 template, and Phusion polymerase (NEB) with 98 °C initial denaturing for 3 min, (98 °C 30 s, 55 °C 30 s, 72 °C 105 s) × 25, and 72 °C final extension for 10 min. PCR product was gel extracted from a 0.7% agarose gel with E.Z.N.A. gel extraction kit (Omega), and recircularized with Gibson assembly^50^. Recircularized plasmid was transformed into DH5α electrocompetent cells and plated on LB agar plates supplemented with tetracycline at 20 µg ml^−1^.

Ptrp promoter through the *cat-upp* was amplified from pRepCM-CAA with primers 5′-GGTGGTAGATCTGTGCACTTCAAAAATCGATG and 5′-GGTGGTGCGGCCGCCAAGCTTCGAATTCTTTATTTCG, adding BglII and NotI sites respectively (underlined), with Phusion polymerase (NEB) with 98 °C initial denaturing for 3 min, (98 °C 30 s, 55 °C 30 s, 72 °C 1 min) × 25, and 72 °C final extension for 10 min. Plasmid pT7wtK-p15A and column purified PCR product (E.Z.N.A. cycle pure kit from Omega) were digested with BglII and NotI (NEB) for 1 h at 37 °C, and gel extracted with E.Z.N.A. gel extraction kit (Omega). 50 ng of pT7wtK-p15A backbone was ligated with three-fold molar excess Ptrp-cat-upp insert with T4 ligase (NEB) for 14 h at 16 °C. Product was transformed into DH5α electrocompetent cells and plated on LB agar plates supplemented with kanamycin at 30 μL ml^−1^. Plasmids were isolated with E.Z.N.A. miniprep kit (Omega) and sequence confirmed. T7 promoter was then deleted using inverse PCR with phosphorylated primers 5′-GTGCACTTCAAAAATCGATG and 5′-GGATCCGTCGACCTGCAG with Phusion polymerase (NEB) with 98 °C initial denaturing for 3 min, (98 °C 30 sec, 55 °C 30 sec, 72 °C 3 min) × 25, and 72 °C final extension for 10 min. Following gel extraction with E.Z.N.A. gel extraction kit (NEB) product was ligated with T4 ligase (NEB) for 14 h at 16 °C, and transformed into DH5α electrocompetent cells and plated on LB agar plates supplemented with kanamycin at 30 μL ml^−1^. Plasmids were isolated with E.Z.N.A. miniprep kit (Omega) and sequence confirmed. This plasmid is named pPtrp-catupp-p15A.

Plasmid pPtrp-p15A (Δcatupp) was prepared from pPtrp-catupp-p15A by PCR with primers 5′-AAGAATTCGAAGCTTGG (forward primer binding at the 3′ end of *cat-upp* gene, including a NotI restriction site in PCR product) and 5′- GCATCAGCGGCCGCAACGCTGCGTAGCAACAGATCTCCTCCTTATGAAAGCGAC (reverse primer binding at 5′ end of gene), adding a BglII/NotI cloning site. Following column purification (E.Z.N.A. cycle pure kit, Omega), product was digested with NotI (NEB), gel extracted (E.Z.N.A gel extraction kit, Omega), and ligated with T4 ligase (NEB) for 14 h at 16 °C. Product was transformed into DH5α electrocompetent cells and plated on LB agar plates supplemented with kanamycin at 30 μL ml^−1^. Plasmids were isolated with E.Z.N.A. miniprep kit (Omega) and sequence confirmed.

Plasmid plpp5-catupp-p15A was prepared from plasmid pPtrp-catupp-p15A and synthesized gBlock (IDT) lpp5-oRBS-BglII (Supplementary Table 1). First, pPtrp-catupp-p15A was amplified with primers 5′-CACTGGATATACCACCGTTG and 5′-GGAAAGCCACGTTGTGTCTC. The linear product is pPtrp-catupp-p15A excluding the Ptrp promoter. Promoter lpp5^53^ with orthogonal ribosome binding site and BglII restriction site^22^ was amplified from gBlock lpp5-oRBS-BglII with primers 5′-GAGACACAACGTGGCTTTCC and 5′-CAACGGTGGTATATCCAGTG. Both PCRs were run with Phusion polymerase (NEB) with 98 °C initial denaturing for 3 min, (98 °C 30 s, 55 °C 30 s, 72 °C 90 sec) × 25, and 72 °C final extension for 10 min. Following gel extraction from 0.7% agarose gel with E.Z.N.A. gel extraction kit (Omega), 50 ng of backbone was recircularized with three-fold molar excess of lpp5-oRBS-BglII insert using Gibson assembly^50^. Product was transformed into DH5α electrocompetent cells, plated in LB plates supplemented with 30 µg ml^−1^ kanamycin, incubated at 37 °C and plasmids isolated and sequenced.

Selection conditions for BL21(DE3)Δupp strain and plasmid system were determined using the pPtrp-catupp-p15A plasmid with the wild-type Shine-Dalgarno sequence (Supplementary Fig. 7a).

Two colonies each of BL21(DE3)Δupp transformed with pPtrp-catupp-p15A (*cat-upp*) or pPtrp-p15A (Δ*cat-upp*) were grown in LB supplemented with kanamycin at 30 μg ml^−1^ at 37 °C overnight with shaking. Fresh LB-kanamycin (30 μg ml^−1^) was inoculated 1/50 with overnight culture and grown for 3 h at 37 °C with shaking. Cultures were normalized to 0.1 OD and 1 μL was plated on (i) M9 minimal media agar plates supplemented with 0.2% casamino acids, 0.4% glucose, 30 µg ml^−1^ kanamycin and 5-fluorouracil at concentrations 0, 0.25, 0.5, 0.75, 1, 2.5, 5, 10, and 50 μg ml^−1^, and (ii) LB-agar plates supplemented with 30 μg ml^−1^ kanamycin and Cm at concentrations 0, 5, 10, 25, 50, 75, 100, 150, and 200 μg ml^−1^. Plates were incubated at 37 °C for 18 h and imaged (Supplementary Fig. 8). We observed robust selection conditions and chose 10 µg ml^−1^ 5-FU for the negative selection (background cell growth ceases by 0.5 µg ml^−1^ when *cat-upp* is expressed under the Ptrp promoter and wild-type SD sequence), and 100 µg ml^−1^ Cm was used for the positive selection (minimum inhibitory concentration 5 µg ml^−1^ for the cells not expressing *cat-upp*). Of note, the Ptrp promoter (medium strength) was used in this initial assay optimization experiments along with a wild-type SD sequence (lower mRNA expression with bigger population of wild-type ribosomes) to more accurately reflect standard orthogonal system conditions (high mRNA expression with lower population of o-ribosomes). Using a stronger promoter at this step, such as lpp5, would result in high cell burden and sickness, and thus give a poor representation of expression levels within the orthogonal system.

For selection of orthogonal pairs, the Shine-Dalgarno site on plasmid plpp5-catupp was fully randomized by PCR mutagenesis using Phusion (NEB), primers 5′-GCATCAAGATCTATGGAGAAAAAAATCACTGG and 5′-CGAGTCCAGATCTNNNNNNNGAAAAAATAACAGATATAGAATTG (IDT), and plpp5-catupp template, with 98 °C initial denaturing for 3 min, (98 °C 30 s, 55 °C 30 s, 72 °C 90 s) × 25, and 72 °C final extension for 10 min. Following DpnI (NEB) digestion for 1 h at 37 °C, PCR product was column purified with E.Z.N.A. cycle pure kit (Omega). Product was digested with BglII (NEB) for 1 h at 37 °C, and purified by gel extraction using E.Z.N.A. gel extraction kit (Omega). Linear product was re-circularized with T4 ligase (NEB) for 14 h at 16 °C.

Ligated product was transformed into DH5α cells (NEB), and plated on LB-agar plates supplemented with 30 µg ml^−1^ kanamycin and incubated overnight at 37 °C. Transformation and plating was repeated until colony counts exceeded 3x library size. Plates were then washed and miniprepped to generate a plasmid library. Two microliters of purified plasmid library was transformed into electrocompetent BL21(DE3)Δupp and plated on M9 minimal media agar plates supplemented with 0.2% casamino acids, 0.4% glucose, 10 µg ml^−1^ 5-FU, 30 µg ml^−1^ kanamycin and 0.1 mM isopropyl-β-D-thiogalactopyranoside (IPTG). Plates were incubated for 24 h at 37 °C. Plates were washed and the pellet was washed three times with LB-Lennox supplemented with 30 µg ml^−1^ kanamycin, and used to inoculate 500 ml LB-Lennox supplemented with 30 µg ml^−1^ kanamycin to prepare electrocompetent cells.

In a first round of selection, the anti-Shine-Dalgarno of pAM552-LT, encoding for wild-type untethered ribosomes, was fully randomized for a library of 4096 theoretical members. Specifically, pAM552-LT ASD was fully randomized by PCR mutagenesis using Phusion (NEB), primers 5′- GCATCAGGTAACCGTAGGGGAACCTGCGGTTGGATCANNNNNNTACCTTAAAGAAGCGTAC and 5′- CCCTACGGTTACCTTGTTACG (IDT), with 98 °C initial denaturing for 3 min, (98 °C 30 s, 55 °C 30 s, 72 °C 2 min) × 25, and 72 °C final extension for 10 min. PCR product was column purified with E.Z.N.A. cycle pure kit (Omega), and digested with BstEII and DpnI (NEB) for 1 h at 37 °C, and purified by gel extraction using E.Z.N.A. gel extraction kit (Omega). Linear product was re-circularized with T4 ligase (NEB) for 14 h at 16 °C. Ligated product was transformed into POP2136 electrocompetent cells, and plated on LB-agar plates supplemented with 50 µg ml^−1^ carbenicillin and incubated overnight at 30 °C. Transformation and plating was repeated until colony counts exceeded 3x library size. Plates were then washed and mini-prepped to generate a plasmid library.

The library was transformed into BL21(DE3)Δupp cells containing the negatively selected mRNA library. Cells were recovered in 1 ml SOC, and used to inoculate 50 ml LB supplemented with 30 µg ml^−1^ kanamycin, 50 µg ml^−1^ carbenicillin and 1 mM IPTG. Cultures were grown for 3 h at 37 °C with shaking at 250 rpm. One ml aliquots were plated on LB agar plates supplemented with 30 µg ml^−1^ kanamycin, 50 µg ml^−1^ carbenicillin, 1 mM IPTG and 100 µg ml^−1^ Cm. Surviving colonies were picked and grown in 96 deep-well format in 750 μL LB media supplemented with 50 μg ml^−1^ carbenicillin and 30 μg ml^−1^ kanamycin at 37 °C for 18 h. Total plasmids were extracted with Zyppy^TM^-96 plasmid miniprep kit (Zymo Research).

To isolate the pAM552-LT rRNA plasmid and plpp5-catupp reporter plasmids from the total plasmid pool, we identified unique restriction sites on each plasmid that is absent from the other (KpnI present on pAM552-LT, BamHI present on plpp5-catupp). To isolate pAM552-LT, we digested the total plasmid pool with BamHI-HF restriction enzyme (NEB), transformed the digestion pool into POP2136 CaCl_2_ chemically competent cells, and plated on LB agar plates supplemented with 50 μg ml^−1^ carbenicillin and grown overnight at 30 °C. To isolate plpp5-catupp, total plasmids were digested with KpnI restriction enzyme (NEB), and transformed into DH5alpha CaCl_2_ chemically competent cells, and plated on LB agar plates supplemented with 30 μg ml^−1^ kanamycin and grown overnight at 37 °C.

Individual plasmids were isolated with E.Z.N.A. miniprep kit (Omega) for sequencing of the Shine-Dalgarno region of plpp5-catupp, and the anti-Shine-Dalgarno region of pAM552-LT (NU genomics core). CaCl_2_ chemically competent BL21(DE3)Δupp cells containing pAM552 plasmid were transformed with the plpp5-catupp isolated members, and plated on LB agar plates supplemented with 50 μg ml^−1^ carbenicillin and 30 μg ml^−1^ kanamycin and grown overnight at 37 °C. Pair performance was initially evaluated by plating cells on a range of Cm. Colonies were picked into 100 μL of LB supplemented with 50 μg ml^−1^ carbenicillin and 30 μg ml^−1^ kanamycin and grown to saturation overnight at 37 °C with shaking. Cultures were diluted 1/50 into fresh LB supplemented with 50 μg ml^−1^ carbenicillin, 30 μg ml^−1^ kanamycin and 1 mM IPTG and grown at 37 °C with shaking for 3 h. LB-agar plates supplemented with 50 μg ml^−1^ carbenicillin, 30 μg mL^−1^ kanamycin, 0.1 mM IPTG and Cm at 0, 0.5, 1, 2.5, 10, 20, 40, 60, 80, 100, 200, 300, 400 or 500 μg mL^−1^ were spot plated with 1 µL of induced culture and incubated at 37 °C for 18 h. Max Cm concentration with growth was noted (Supplementary Fig. 9a).

Reporter plasmids from top performing pairs were pooled and transformed into fresh BL21(DE3)Δupp strain. Cells were plated on LB agar plates supplemented with 30 μg mL^−1^ kanamycin and grown overnight at 37 °C. Plates were washed and the pellet was washed three times with LB-Lennox supplemented with 30 µg mL^−1^ kanamycin, and used to inoculate 500 ml LB-Lennox supplemented with 30 µg mL^−1^ kanamycin to prepare electrocompetent cells.

With version 2 tethers evolved and characterized, the improved tether sequences were cloned into poRibo-T2 plasmid^22^, named pORT1A. The anti-Shine-Dalgarno sequence of pORT1A was randomized with the protocol described above, and passaged through POP2136 cells at 30 °C (expression from p_LT_ promoter repressed by cI repressor). Positive selection was repeated as described above in the first round. Total plasmid was extracted from colonies using the Zyppy^TM^-96 plasmid miniprep kit (Zymo Research). Reporter and rRNA plasmids were isolated with KpnI and BamHI-HF digestion, respectively, as before.

### Evaluation of new orthogonal pairs

Plasmid plpp5.A.cat was prepared by digesting plasmid plpp5.A.gfp with BglII (NEB) and NotI (NEB), restriction sites flanking the *sf-gfp* coding sequence. Backbone was purified by gel extraction using E.Z.N.A. gel extraction kit (Omega). PCR was performed on template pAM552C (Mankin Lab) using primers 5′-GGTGGTAGATCTATGGAAAAAAAAATCACCGG and 5′-GGTGGTGCGGCCGCGCTTATTAGGCGGGCTAGG (BglII and NotI restriction sites underlined) with Phusion polymerase (NEB) with 98 °C initial denaturing for 3 min, (98 °C 30 s, 55 °C 30 s, 72 °C 2 min) × 25, and 72 °C final extension for 10 min.

For the superfolder green fluorescent protein (*sf-gfp*) assay, three colonies for each pair were picked and grown to saturation at 37 °C. Fresh LB supplemented with 30 µg ml^−1^ kanamycin, 50 µg mL^−1^ carbenicillin and 1 mM IPTG was inoculated with 1/50 saturated culture and grown at 37 °C for 18 h on Biotek Synergy H1 plate reader with linear shaking at 2 mm. OD_600_ and 485/528 excitation/emission were monitored.

For Cm acetyltransferase (CAT) assay, six colonies for each pair were picked and grown to saturation at 37 °C. Fresh LB supplemented with 30 µg mL^−1^ kanamycin, 50 µg mL^−1^ carbenicillin and 1 mM IPTG was inoculated with 1/50 saturated culture and grown at 37 °C for 3 h. Ninety-six-well plates containing 100 µL LB supplemented with 30 µg mL^−1^ kanamycin, 50 µg mL^−1^ carbenicillin and 1 mM IPTG, and 0, 0.5, 1, 2.5, 5, 10, 20, 30, 40, 50, 60, 70, 100, 150, 200 or 300 µg mL^−1^ Cm were inoculated with 1/100 induced culture. Plates were incubated for 18 h at 37 °C with shaking. OD_600_ was read on BioTek Synergy H1 plate reader, and IC_50_ values (Fig. 5c) determined using the IC_50_ toolkit (ic50.tk).

### Evaluation of Ribo-T growth in minimal media

Wild type ribosomes, Ribo-T v1, and Ribo-T v2 growth (both orthogonal and non-orthogonal) were grown on M9-casamino acids (M9CA) minimal media plates at 30, 37, and 42 °C in a spot-plating format. Colonies appeared after 24–48 h incubation on each plate, and were subsequently picked and used to inoculate a Costar flat bottom 96-well plate containing 100 μL of M9CA supplemented with 50 μg mL^−1^ carbenicillin (orthogonal constructs) or 50 μg mL^−1^ carbenicillin and 1 mg mL^−1^ erythromycin. Growth rates were monitored at 30, 37, and 42 °C in a BioTek microplate reader. Absorbance at 600 nm was read every 10 min (continuous linear shaking with a 2-mm amplitude). Doubling times were calculated from the growth curve readings during logarithmic growth as determined by regression.

### Expression of recombinant proteins using oRibo-T v2

Plasmids plpp5.B.LacZ and plpp5.B.ApNGT were prepared by Gibson assembly. Briefly, PCR products were digested with DpnI (NEB), gel extracted as before, and Gibson assembled^50^ with 50 ng backbone and three-fold molar excess insert. Two μL of the assembled products were co-transformed with Ribo-T v2 (pORT3) into BL21(DE3) cells via electroporation, recovered in 1 ml SOC, and plated on LB-agar supplemented with 50 μg mL^−1^ carbenicillin and 30 μg mL^−1^ kanamycin. Plates were grown overnight at 37 °C. Plasmids were purified from colonies with E.Z.N.A. miniprep kit (Omega), and sequence-confirmed (Northwestern Sequencing Core).

Sequence confirmed clones were grown overnight in 5 mL LB supplemented with 50 μg mL^−1^ carbenicillin and 30 μg mL^−1^ kanamycin. After ~18 h, saturated cultures were used to inoculate fresh cultures of 5 mL LB supplemented with 30 µg ml^−1^ kanamycin, 50 µg mL^−1^ carbenicillin. At an OD_600_ of 0.8, the cultures were induced with 1 mM IPTG. Cultures were grown for 3 h at 37 °C with shaking at 250 rpm. Ten microliters of each expression culture was analyzed by SDS-PAGE, on a 4–15% gradient polyacrylamide gel (BioRad) and stained with Coomassie. Band intensities were subsequently quantified using Image Studio software.

### Purification of recombinant proteins

A sequence confirmed clone containing oRibo-T v2 and the orthogonal protein construct of interest was used to inoculate a 5 mL overnight culture in LB supplemented with 50 μg mL^−1^ carbenicillin and 30 μg mL^−1^ kanamycin. After ~18 h, saturated cultures were used to inoculate fresh cultures of 5 ml LB supplemented with 30 µg mL^−1^ kanamycin, 50 µg mL^−1^ carbenicillin. At an OD_600_ of 0.8, the cultures were induced with 1 mM IPTG. Cultures were grown for 3 h at 37 °C with shaking at 250 rpm. The induced cultures were pelleted at 6170 × *g* for 10 min at 4 °C. The pellets were washed with in 10 mL of binding buffer (50 mM NaH_2_PO_4_, 300 mM NaCl, 10 mM Imidazole, 6 mM BME, adjusted to pH 8) and stored at −20 °C. Cells were resuspended in cold lysis buffer (1 M Tris HCl, pH 8, 3 M NaCl, 50% Glycerol, 1 M BME) with vortexing and rocking. The cell suspension was cooled on ice for 10 min and then sonicated in 10 bursts of 10 s followed by intervals of 10 sec of cooling. Cellular debris was removed by two centrifugations at 4 °C for 15 min at 14,500 × *g* in SS-34 centrifuge tubes. The lysate was collected after the second centrifugation, and transferred to Ni-NTA resin that was pre-equilibrated with three column volumes of binding buffer. Proteins were purified in batch, in 15 mL falcon tubes. The lysate was incubated with the Ni-NTA resin for one hour at 4 °C with rocking. After incubation, the resin was centrifuged at 500 × *g* for 5 min, and the supernatant was removed. The Ni-NTA resin was washed three times with three column volumes of binding buffer, and then incubated with elution buffer (50 mM NaH_2_PO_4_, 300 mM NaCl, 200 mM Imidazole, 6 mM BME, adjusted to pH 8). Elution fractions were collected, run on an SDS-PAGE gel, and stained with Coomassie.

### Strain construction for ncAA incorporation with oRibosomes

Strain C321.ΔA^32^ contains the cI repressor, which represses p_L_ promoter driving expression of the rRNA constructs. Therefore, the strain was prepared for use in the following experiments. Firstly, mutS^−^ genotype was mutated back to mutS wild-type (mutS^+^) by multiplex advanced genome engineering (MAGE)^54^ and the MAGE oligo accccatgagtgcaatagaaaatttcgacgcccatacgcccatgatgcagcagtatctcaggctgaaagcccagcatcccgagatcctgc. Mutations to mutS^+^ were screened with colony PCR and primers 5′-CATGATGCAGCAGTATCTCAG and 5′-CTTCTGCATACAGCAGTTC and confirmed by sequencing.

To remove cI repressor, the λ-red machinery and the *bla* resistance marker, a kanamycin knockout cassette was generated from pKD4 plasmid^51^ with primers 5′-GTATGTCGTTTCAGCTAAACGGTATCAGCAATGTTTATGTAAAGATGTGTAGGCTGGAGCTGCTTC and 5′-TTTGCCGACTACCTTGGTGATCTCGCCTTTCACGTAGTGGACAAAGTCCATATGAATATCCTCCTTAG with Phusion polymerase and 98 °C initial denaturing for 3 min, (98 °C 30 s, 55 °C 30 s, 72 °C 30 s) × 25, and 72 °C final extension for 10 min. Product was column purified with E.Z.N.A. cycle pure kit (Omega). Expression of λ-red machinery was induced with a 15 min incubation at 42 °C, and electrocompetent cells were prepared. KanR knockout cassette was electroporated into the cells, plated on LB agar supplemented with 30 μg mL^−1^ kanamycin and incubated overnight at 42 °C to select against heat-induced toxic expression of λ-red cassette. Kanamycin-resistant colonies were screened for sensitivity to carbenicillin, indicating loss of *bla*. A sensitive colony was picked and transformed with pCP20 plasmid for removal of kanamycin marker by the incorporated flippase sites^51^. Transformed cells were plated on LB agar supplemented with 50 μg mL^−1^ carbenicillin and grown overnight at 30 °C. Colonies were picked, plated on LB agar plates, and grown overnight at 42 °C to select for loss of pCP20 plasmid. Colonies were checked for kanamycin sensitivity, and deletion was confirmed by sequencing of PCR product from colony PCR using primers 5′-GCCGACTCTATATCTATACCTTCATC and 5′-GCAACCGAGCGTTCTGAAC, and Multiplex PCR mix (Qiagen), flanking the deletion site. Furthermore, this strain has *upp* gene knocked out using the same methodology described in preparing the BL21(DE3)Δupp strain above. This strain is named MCJ.1217.

### Combined orthogonal ribosome-sf-gfp reporter system

The orthogonal *sf-gfp* cassette was amplified from plpp5.B.gfp template with primers 5′-AGAGTTGGATCCCCTTGTATTACTGTTTATGTAAGC and 5′-AAGAGTT**GGCGCGCC***AAAAAAAAGCCCGCCTTTCGGCGGGCTTTG*TTATTTTTCGAACTGCGGATG for forward orientation, and primers 5′-AGAGTT**GGCGCGCC**CCTTGTATTACTGTTTATGTAAGC and 5′-AAGAGTTGGATCC*AAAAAAAAGCCCGCCTTTCGGCGGGCTTTG*TTATTTTTCGAACTGCGGATG for reverse orientation using Phusion polymerase (NEB) with 98 °C initial denaturing for 3 min, (98 °C 30 s, 55 °C 30 s, 72 °C 2 min) × 25, and 72 °C final extension for 10 min. Added BamHI restriction site is underlined, added AscI restriction site is bolded, and t500 terminator is italicized. Plasmid backbones were amplified from plasmids pAM552, pO2 or pORT3 with primer 5′-CCTGTCGTCATATCTACAAG flanking the AscI restriction site and primer 5′-AAGAGTTGGATCCTGTAGAAACGCAAAAAGGCCATC, adding in a BamHI restriction site, using Phusion polymerase (NEB) with 98 °C initial denaturing for 3 min, (98 °C 30 s, 55 °C 30 s, 72 °C 2 min) × 25, and 72 °C final extension for 10 min. PCR products were individually purified by E.Z.N.A. cycle pure kit (Omega), and digested with BamHI-HF and AscI (NEB) for 1 h at 37 °C. Digestion products were purified by gel extraction with 1% agarose gel and E.Z.N.A. gel extraction kit (Omega), and ligated in all combinations (Supplementary Fig. 13a) with T4 DNA ligase (NEB). Two μL of each ligation product was transformed into POP2136 cells via electroporation, plated on LB-agar supplemented with 50 μg ml^−1^ carbenicillin, and grown overnight at 30 °C to repress plasmid *rrn* expression. Plasmids were purified from colonies with E.Z.N.A. miniprep kit (Omega), and sequence-confirmed (Northwestern Sequencing Core). Plasmids constructed are named pAM.B.gfp-f, pAM.B.gfp-r, pO2B.gfp-f, pO2B.gfp-r, pORT3B.gfp-f, and pORT3B.gfp-r.

Six replicates of each construct were picked and grown to saturation at 30 °C in LB supplemented with 50 µg ml^−1^ carbenicillin. Fresh LB supplemented with 50 µg ml^−1^ carbenicillin, was inoculated with 1/50 volume saturated culture and grown at 30 °C for 4 h, then 42 °C for 12 h in the Biotek Synergy H1 plate reader with linear shaking at 2 mm. OD_600_ and fluorescence (485 nm/528 nm excitation/emission) was monitored.

### Incorporation of p-azido-phenylalanine using oRibo-T

The integrated ribosome-*sf-gfp* plasmids with the *sf-gfp* gene in the reverse direction relative to rRNA operons were used as the backbone, and amplified with primers 5′-GACCACATGGTTCTGCAC and 5′-CGCTGAATTTGTGACCGTTC with the same PCR conditions as above. Plasmids pDT7sfGFP1TAGTT2 (1TAG) and pDT7sfFP5TAGTT2 (5TAG)^45^ were used as templates with primers 5′-CGGTCACAAATTCAGCGTG and 5′-TTCGTGCAGAACCATGTG with the same PCR conditions as above. PCR products were digested with DpnI (NEB), gel extracted as before, and Gibson assembled^50^ with 50 ng backbone and three-fold molar excess insert. Two μL of the assembled products were transformed into POP2136 cells via electroporation, plated on LB-agar supplemented with 50 μg ml^−1^ carbenicillin, and grown overnight at 30 °C. Plasmids were purified from picked colonies with E.Z.N.A. miniprep kit (Omega), and sequence-confirmed (Northwestern Sequencing Core).

Plasmid pEVOL-pAzF, a gift from Peter Schultz (Addgene plasmid # 31186)^41^, and the sequence-verified plasmid of each ribosome-*sf-gfp* construct were co-transformed into MCJ.1217 cells and plated on LB agar plates supplemented with 50 μg ml^−1^ carbenicillin and 34 μg ml^−1^ Cm. Six colonies each were picked and grown to saturation at 37 °C in LB supplemented with 50 µg ml^−1^ carbenicillin and 34 µg ml^−1^ Cm. Fresh LB supplemented with 50 µg ml^−1^ carbenicillin, 34 µg ml^−1^ Cm, 0.2% w/v arabinose, 1 mM IPTG, and 1 mM *p*-azido-ʟ-phenylalanine was inoculated with 1/50 volume saturated culture and grown at 37 °C for 18 h in the Biotek Synergy H1 plate reader with linear shaking at 2 mm. OD_600_ and fluorescence (485 nm/528 nm excitation/emission) was monitored.

### Chemical probing of the structure of the Ribo-T tethers

Ribo-T v1 and Ribo-T v2 were isolated from the exponentially-growing E. coli SQ171fg cultures^22^. Specifically, cells expressing Ribo-T v1 or Ribo-T v2 were grown overnight at 37 °C in LB medium supplemented with 50 μg ml^−1^ ampicillin and 25 µg ml^−1^ spectinomycin. The cultures were diluted 1:100 into 1 L of fresh LB media supplemented with the same antibiotics and grown with vigorous shaking until optical density reached A_600_ = 0.5. Cells were collected by centrifugation for 20 min at 4000 rpm (4 °C) in JA-10 rotor (Beckman) and stored at −80 °C. Cell pellets were resuspended in 20 ml of the buffer 10 mM HEPES-KOH, pH 7.6, 50 mM KCl, 10 mM Mg(OAc)_2_, 7 mM β-mercaptoethanol and lysed in EmulsiFlex-C3 homogenizer (AVESTIN Inc) at 15000 psi for 5 min. Lysate was clarified by centrifugation for 30 min at 20,000 × *g* (4 °C) in JA-25.50 rotor (Beckman). After adding (NH_4_)_2_SO_4_ to 1.5 M, tubes were centrifuged for 1 h at 20,000*×g* (4 °C) in JA-25.50 rotor (Beckman). The Ribo-T-containing supernatant was filtered through the 0.22-µm ∅ 30 mm polyethersulfone (PES) membrane filter, (CELLTREAT scientific products) and loaded on the 5 ml HiTrap Butyl FF column (GE Healthcare Life Sciences) in the buffer 20 mM HEPES-KOH, pH 7.6, 10 mM Mg(OAc)_2_, 7 mM β-mercaptoethanol, 1.5 M (NH_4_)_2_SO_4_. The column was washed with 25 ml of 20 mM HEPES-KOH, pH 7.6, 10 mM Mg(OAc)_2_, 7 mM β-mercaptoethanol, 1.2 M (NH_4_)_2_SO_4_, and Ribo-T were eluted with the buffer 20 mM HEPES-KOH, pH 7.6, 10 mM Mg(OAc)_2_, 7 mM β-mercaptoethanol, 0.75 M (NH_4_)_2_SO_4_. Eluate fractions containing Ribo-T were pooled together and loaded over 16 ml of 30% sucrose cushion prepared in the buffer 20 mM HEPES-KOH, pH 7.6, 10 mM Mg(OAc)_2_, 30 mM NH_4_Cl, 7 mM β-mercaptoethanol. Ribosomes were pelleted by centrifugation at 36,000 rpm for 18 h at 4 °C in the Type 70 Ti rotor (Beckman). Ribosome pellets were resuspended in the storage buffer (20 mM HEPES-KOH pH 7.6, 30 mM KCl, 6 mM Mg(OAc)_2_, 7 mM β-mercaptoethanol) and stored at −80 °C.

Modification of Ribo-T with dimethylsulfate (DMS) was carried out in the final volume of 50 µl of the buffer 80 mM HEPES/KOH pH 7.6, 10 mM MgCl_2_, 100 mM NH_4_Cl; containing Ribo-T at the concentration of 0.2 µM. The modification reaction was initiated by addition of 2 µl of DMS (diluted 1:10v/v in ethanol) to the Ribo-T solution. Reactions were incubated for 10 min at 37 °C and quenched by addition of 50 µl of stop buffer (300 mM NaAc, pH 5.5, 500 mM β-mercaptomethanol) and 300 µl ethanol.

rRNA was isolated by phenol-chloroform extraction and used as a template for primer extension reactions, employing primer L2904 with the AAGGTTAAGCCTCACGG for the T1 linker and primer S1516 with the CCCTACGGTTACCTTGTTACG for the T2 linker.

### RNA structure modelling

RNA structure prediction and analysis software (RNAStructure)^29^ was used to compare the secondary structure and hybrid free energies of the Ribo-T v2 tethers. Specifically, the bifold web server was used to compare the hybrid free energy folds between the two sequences of RNA, also allowing potential intramolecular base pairing interactions. Default parameters of 5% maximum energy difference, maximum loop size of 30 nucleotides, and a temperature of 310.15K were set before query submission. Structures with the lowest hybrid free energy were selected for comparison across library members.

### Protein secondary structure map generation

Protein secondary structure maps were generated using STRIDE^55^, a web server for secondary structure assignment. PDB identifiers for each protein were input to the webserver from RCSB PDB. Visual outputs were produced from each protein. The following PDB IDs were used: 1EMA [https://www.ncbi.nlm.nih.gov/pubmed/8703075], 3CLA [https://www.ncbi.nlm.nih.gov/pubmed/2187098], 3Q3E [https://www.ncbi.nlm.nih.gov/pubmed/21908603], and 1JYX [https://www.ncbi.nlm.nih.gov/pubmed/11732897].

### Reporting summary

Further information on research design is available in the Nature Research Reporting Summary linked to this article.

## Supplementary information

Supplementary Information

Reporting Summary

Source Data

## Data Availability

The following PDB IDs were used in this study: 1EMA [https://www.ncbi.nlm.nih.gov/pubmed/8703075], 3CLA [https://www.ncbi.nlm.nih.gov/pubmed/2187098], 3Q3E [https://www.ncbi.nlm.nih.gov/pubmed/21908603], and 1JYX [https://www.ncbi.nlm.nih.gov/pubmed/11732897]. The authors declare that all data generated in this study are included in this article and its supplementary information files. The source data underlying Figs. 2d and 3b–d, 5b, c, 6b, and Supplementary Figs. 2, 4a, b, 5a–f, 6, 8a, b, 9a, 10a, b, 11a–c, 12a, b, 13b, d are provided as a Source Data file. All other relevant data are available from the authors upon reasonable request.
