# Supplementary material for: Engineered ribosomes with tethered subunits for expanding biological function
Source: Nat Commun. 2019 Sep 2;10:3920. doi: 10.1038/s41467-019-11427-y (PMC6718428; doi:10.1038/s41467-019-11427-y)
Supplement: Supplementary file 1 — Supplementary Information [file 41467_2019_11427_MOESM1_ESM.pdf]

Engineered ribosomes with tethered subunits for expanding biological function

Supplementary Figures and Tables

Carlson et al.

Supplementary Figures

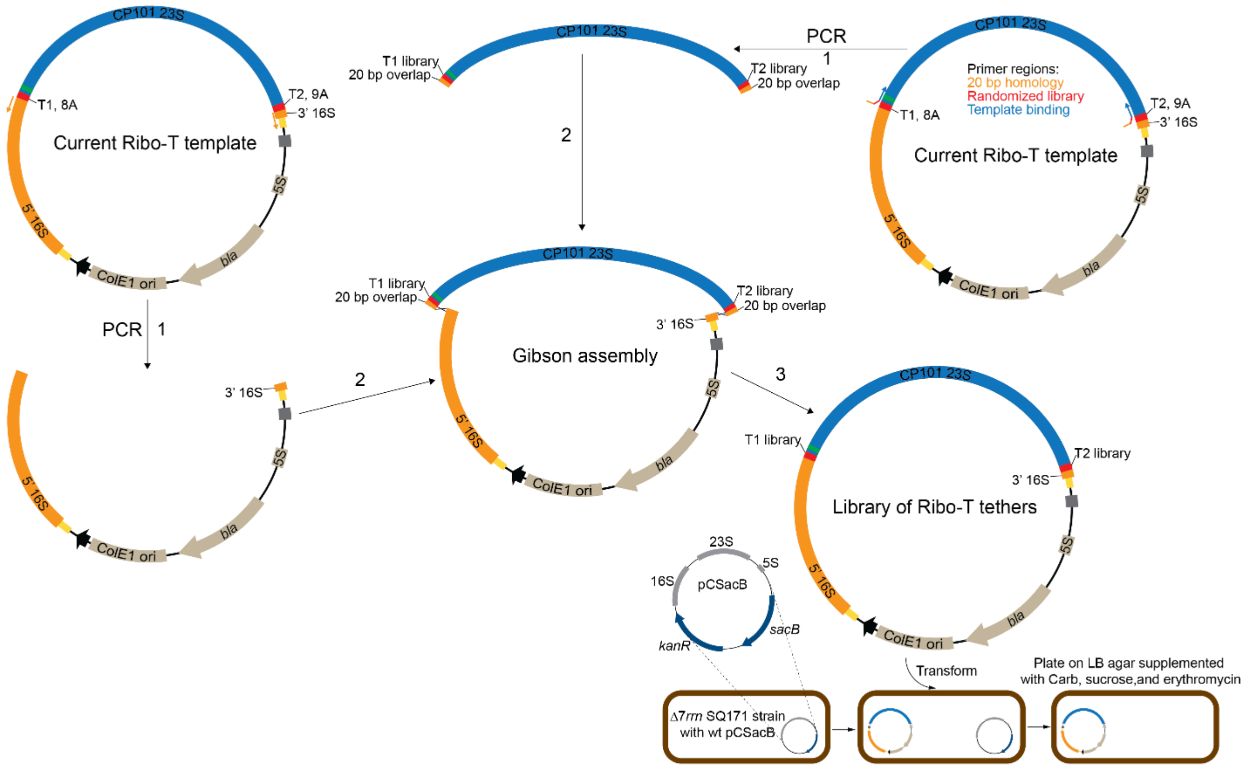

Supplementary Figure 1. Library construction and selection strategy for tether optimization. Plasmid backbone is generated by PCR with primers excluding the circularly permuted 23S at Helix 101 (CP101 23S), shown on the left. In a separate PCR reaction, the tether libraries for tethers T1 and T2 are added to the CP101 23S segment with respective primers from Supplementary Table 1, with added homology to the generated backbone (top right and top middle). Plasmids with randomized libraries are generated from these pieces with Gibson assembly (middle). Tether libraries are transformed into SQ171 strains (Δ7*rrn*) and pCSacB maintenance plasmid is replaced, resulting in cells growing exclusively with Ribo-T tether library members (bottom right).

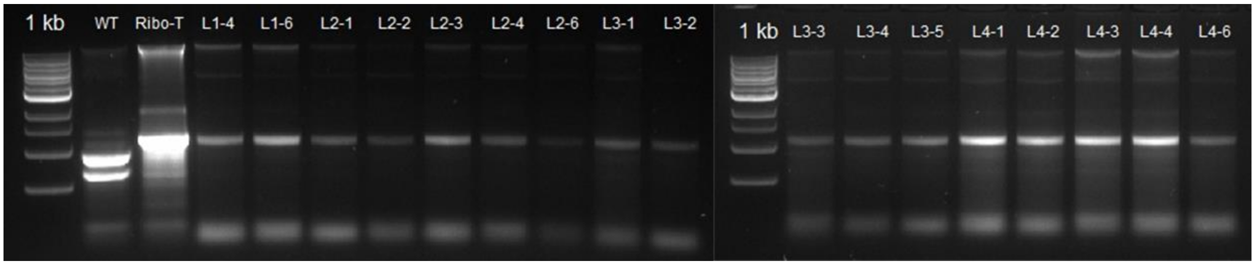

Supplementary Figure 2. Total RNA gel of selected library members. WT: wildtype ribosomes extracted from SQ171fg cells with pAM552 plasmid. Sample names are organized as LX-Y; where X is the library number (1-4), and Y is a clone number from that library.

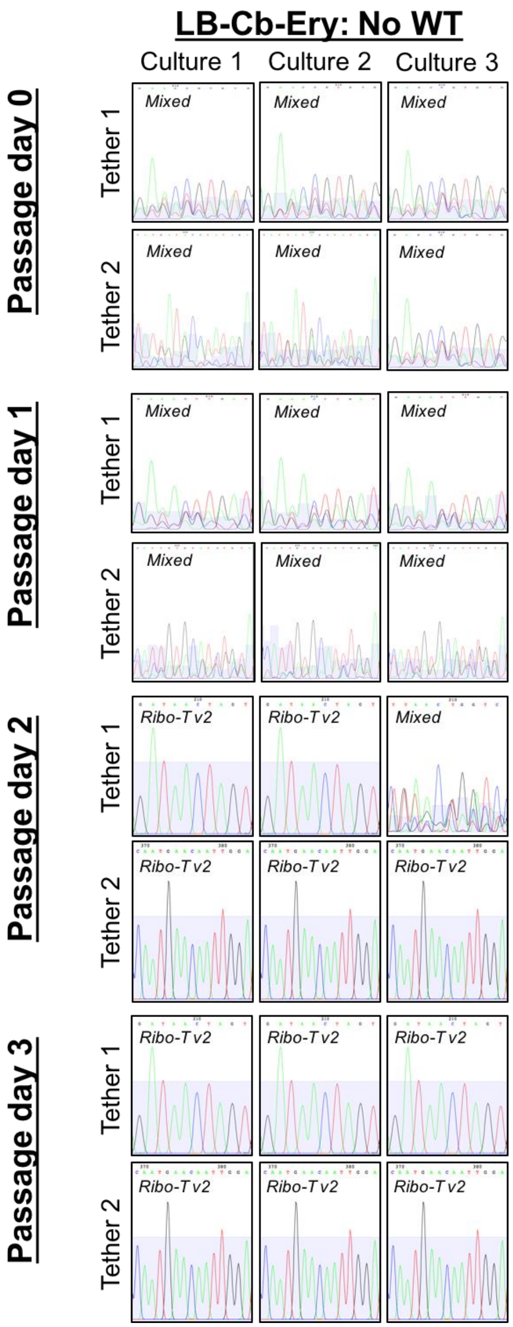

Supplementary Figure 3. Liquid culture enrichment and competition assay shows Ribo-T v2 as the dominant genotype following multiple generations of growth. The top 15 Ribo-T winners identified were co-cultured in triplicate and passaged for 3 days. Between each passage, the bulk populations from the culture were sequenced and analyzed.

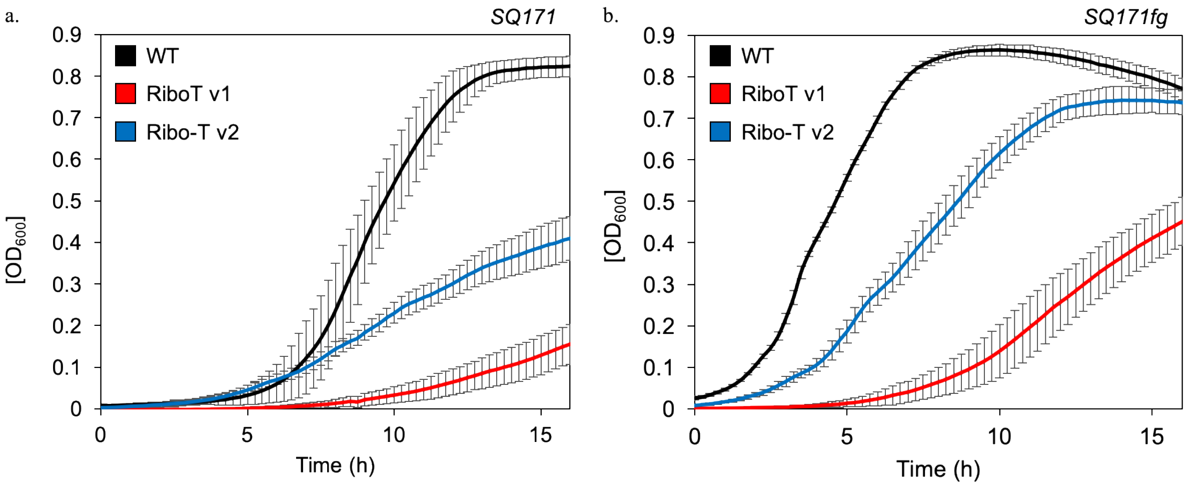

Supplementary Figure 4. Ribo-T v2 improvements enhance growth of SQ171 and SQ171fg cells in which Ribo-T replaces wild-type ribosomes. Growth curves of (a) SQ171 cells (Error bars = 1 SD; n=5). and (b) SQ171fg cells (Error bars = 1 SD; n=3) transformed with pAM552 DNA (wild-type, black), or Ribo-T v1 (red) or Ribo-T v2 (blue).

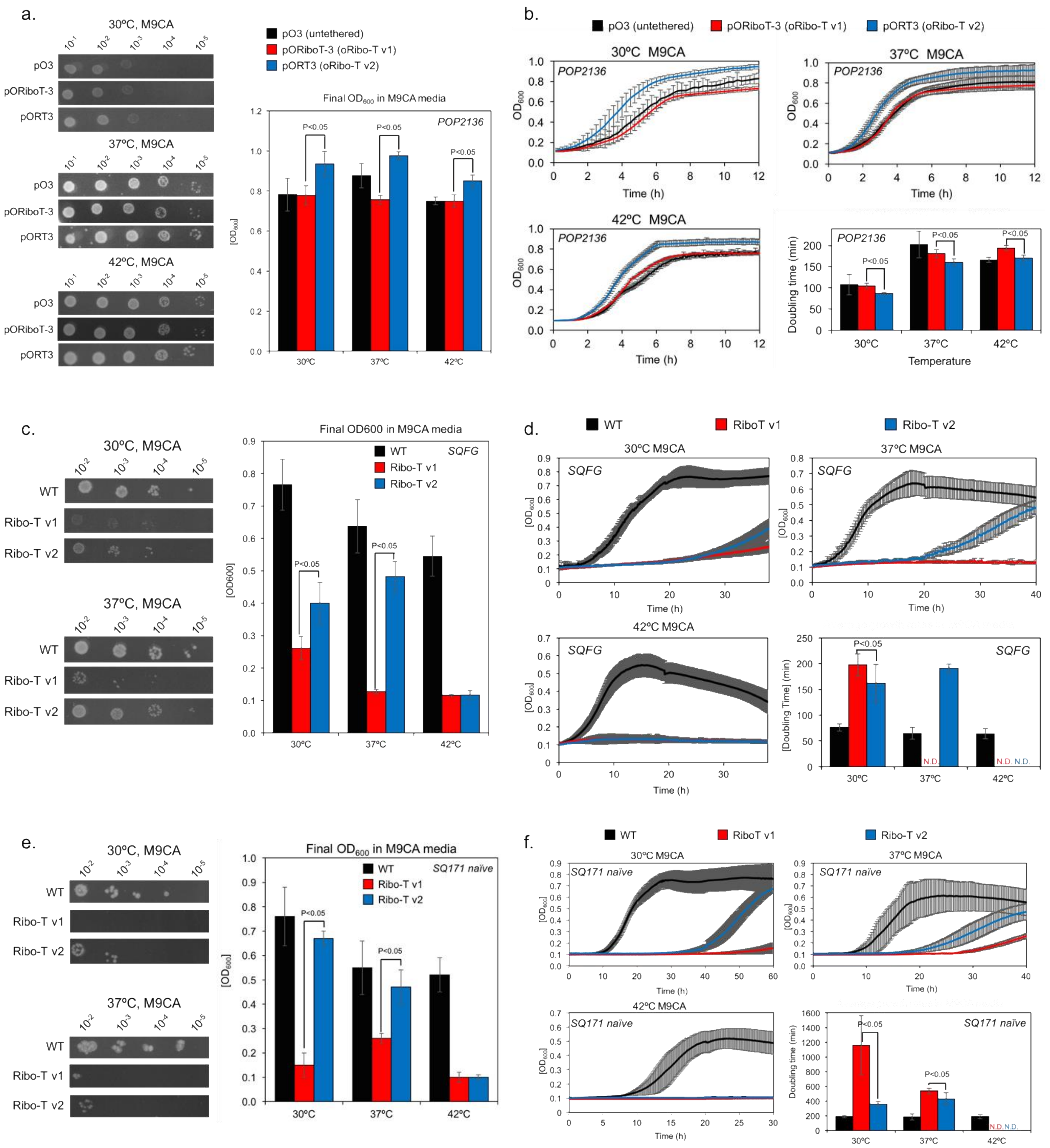

Supplementary Figure 5. Ribo-T v2 enhances cell fitness as compared to Ribo-T v1 when grown on minimal media (M9 casamino acids, M9CA) at various temperatures in different cell backgrounds. a. Orthogonal and non-orthogonal untethered ribosomes, Ribo-T v1, and Ribo-T v2, were grown in and on M9CA media in a-b. POP cells (n=6-8), c-d. SQ171fg cells (n=5-6), and e-f. SQ171 cells (n=6). Final OD_{600} and doubling times were analyzed. Error bars = 1 SD.

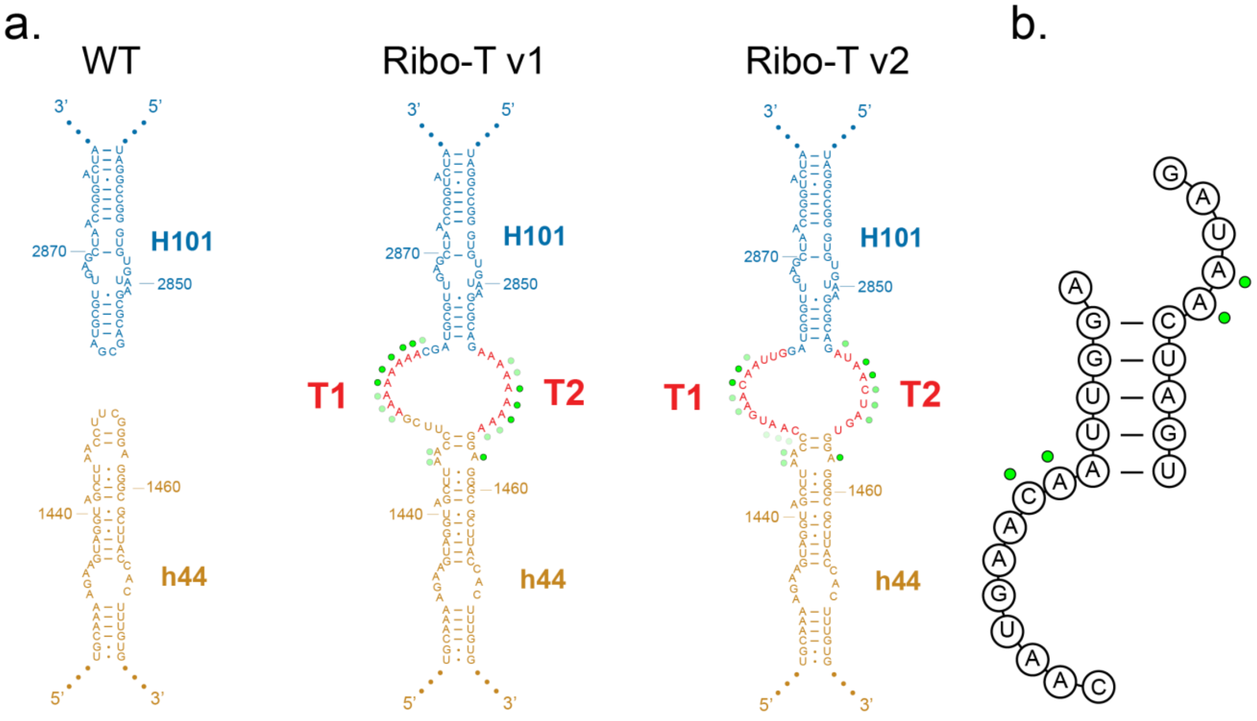

Supplementary Figure 6. Chemical probing and prediction of the structure of the Ribo-T v2 linkers. a. The diagrams represent the structures of helices H101 and h44 in wild-type ribosomes (left), Ribo-T v1 (middle), and Ribo-T v2 (right), with the nucleotide accessible to the dimethyl sulfate (DMS modification indicated by green dots). Highly accessible residues are indicated by brightly-colored dots b. The predicted secondary structure formed by the Ribo-T v2 tethers with the nucleotides most accessible for DMS modifications indicated.

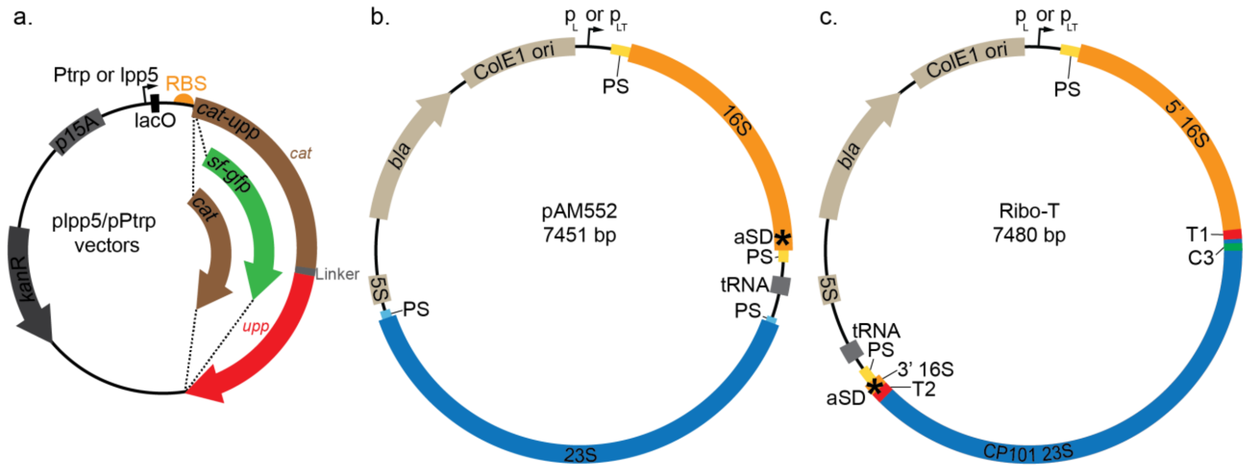

Supplementary Figure 7. Reporter plasmid and ribosome plasmid maps. a. Reporter plasmid with Ptrp or lpp5 promoter, and chloramphenicol acetyltransferase-uracil phosphoribosyltransferase (*cat-upp*), super folder green fluorescent protein (*sf-gfp*) (reporter plasmid: plpp5.gfp), or *cat* gene (reporter plasmid: plpp5.cat). Plasmid contains kanamycin resistance and p15A origin of replication. b. Plasmid coding for untethered ribosomal RNA (rRNA) from *rrnb* operon, with either p_L promoter or p_{LT} promoter for orthogonal expression. Plasmid contains ampicillin resistance gene lactamase, and ColE1 origin of replication. rRNA processing stems (PS) and anti-Shine-Dalgarno (aSD) sites indicated. c. Plasmid coding for tethered ribosome Ribo-T. 5’ tether 1 (T1) and 3’ tether 2 (T2), and 23S circular permutation connecting piece C3 noted (plasmid name: pRibo-T vX; where X is either 1 or 2).

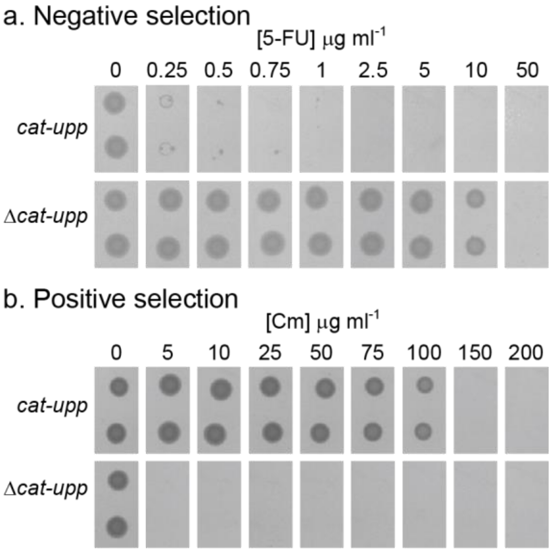

Supplementary Figure 8. Combined positive and negative selection scheme for evolving new orthogonal Shine-Dalgarno/anti-Shine-Dalgarno pairs. a. BL21(DE3)Δupp cells with pPtrp-catupp-p15A and *cat-upp* expressed die in the presence of 5-Fluorouracil. b. BL21(DE3)Δupp cells with pPtrp-catupp-p15A and *cat-upp* expressed gain resistance to the antibiotic chloramphenicol. Plates shown are representative of at least 3 independent experiments.

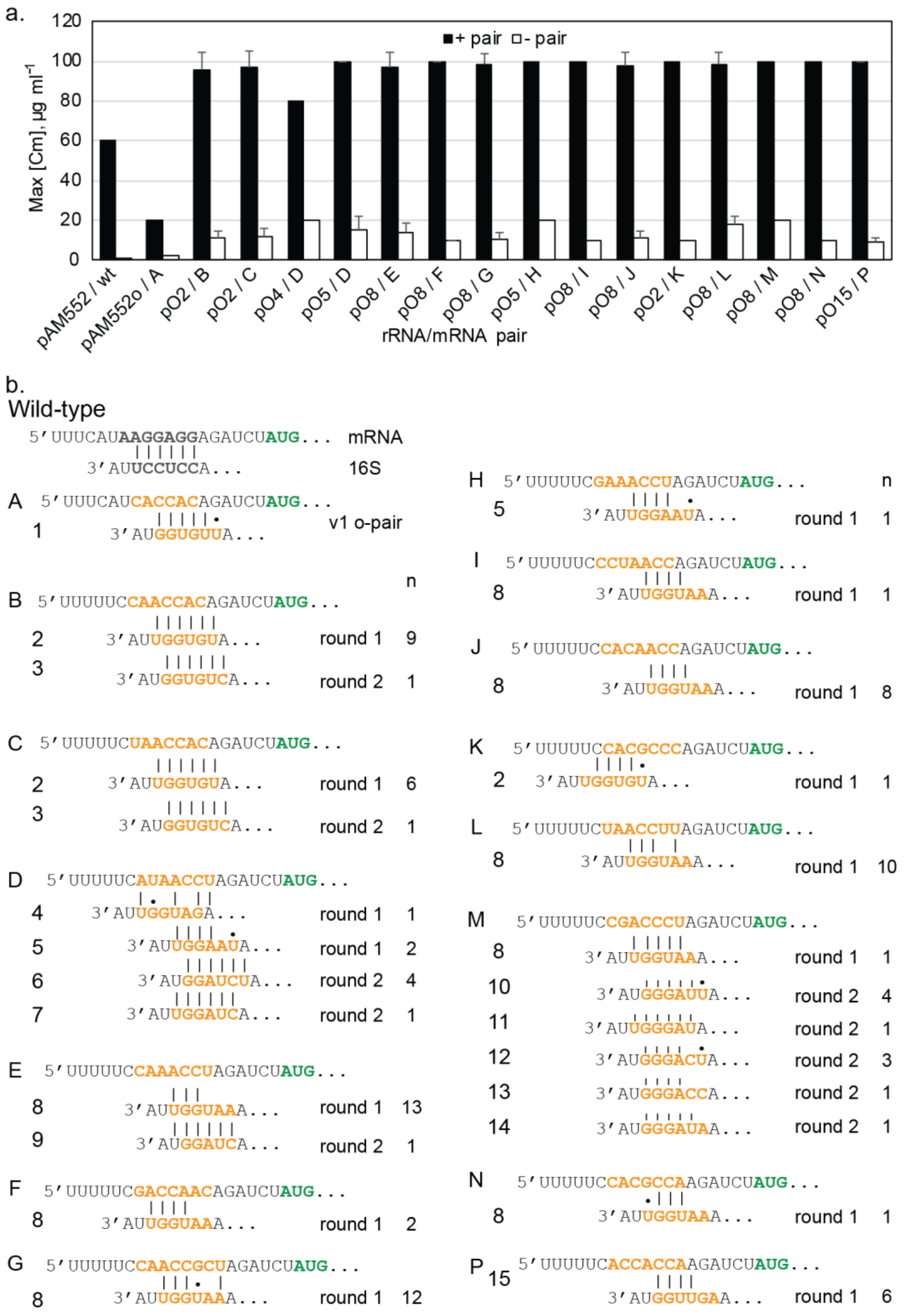

Supplementary Figure 9. Evolved orthogonal pairs. a. Activity testing of untethered orthogonal ribosomes and mRNA following first round of positive selection. Controls: pAM552/wt: wild-type SD expression. pAM552o/A, original orthogonal system in untethered context. b. Selected Shine-Dalgarno (SD) and anti-SD sequences. Round of selection and number of colonies (n) noted for each pair sequence. Orthogonal mRNA SD sequence is labeled with letters, and orthogonal 16S aSD sequence is labeled with numbers. Pair 1A is the previously published orthogonal pair noted as “v1 o-pair”. Error bars = 1 SD for pairs possessing n>1 colonies.

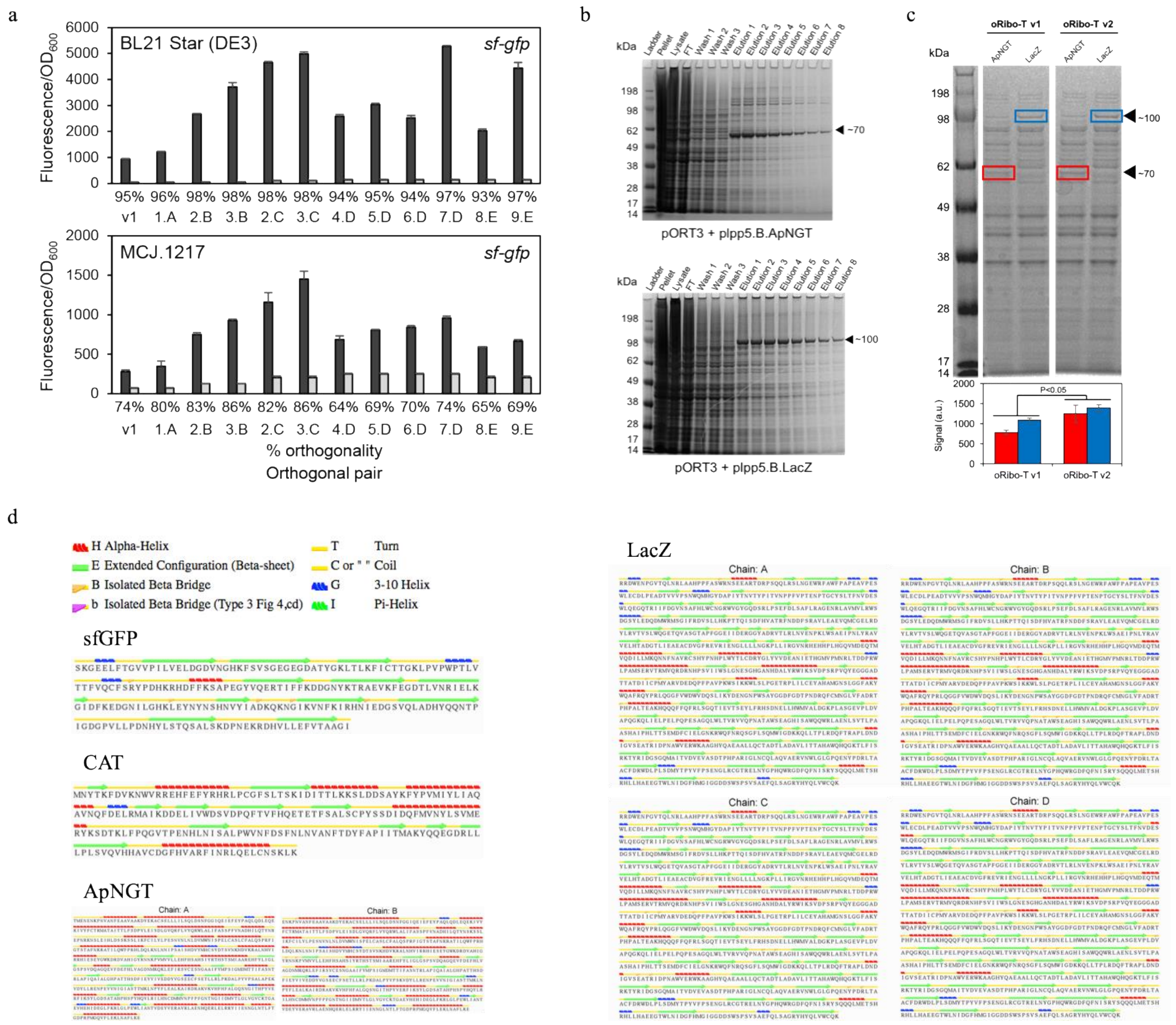

Supplementary Figure 10. Orthogonal Ribo-T v2 is capable of synthesizing diverse proteins of different sizes, compositions, and functions. a. Orthogonal expression of *sf-gfp* in BL21 Star (DE3) strain (top panel). Orthogonal expression of *sf-gfp* in C321.ΔA derived strain MCJ.1217 (bottom panel). MCJ1217 (C321.ΔA.mutS$^+$.Δλred.Δupp) is a variant of the fully recoded C321.ΔA strain [27,28], and provides benefits for non-canonical amino acid incorporation using amber suppression. b. SDS PAGE gels of the expression and purification of non-model proteins. c. SDS-PAGE expression gel comparison between oRibo-T v1 and oRibo-T v2. oRibo-T v2 had a 37% improved expression level of LacZ (n=3, paired t-test, 2 df) and a 22% improved expression level of ApNGT (n=3, paired t-test, 2 df) over oRibo-T v1. Error bars = 1 SD. Gel is representative of three independent replicates (n=3). d. The secondary structure maps of each protein.

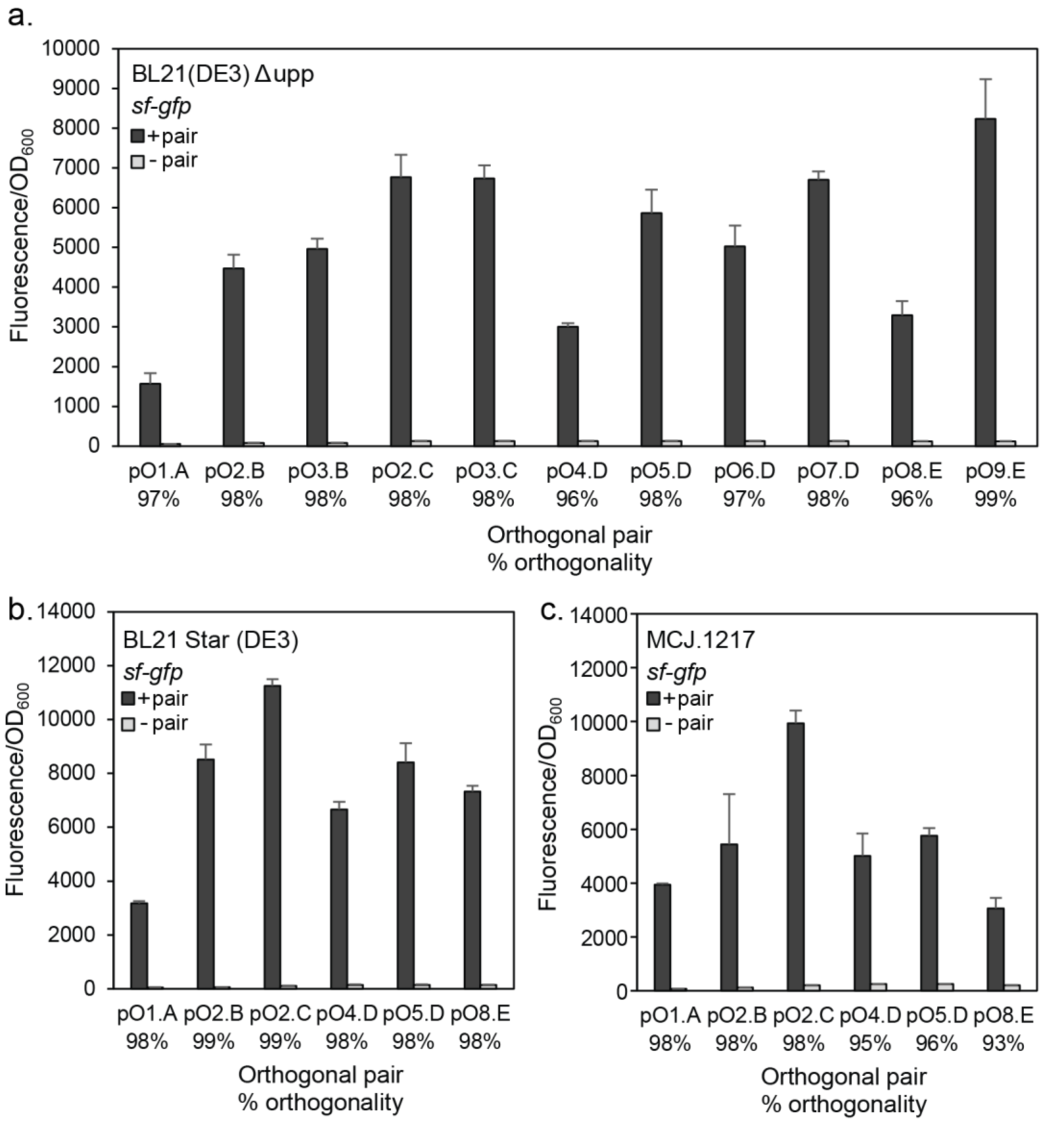

Supplementary Figure 11. Orthogonal pair activity in untethered ribosomes in BL21(DE3)Δupp strain. Orthogonal pair activity in untethered ribosomes in BL21 Star (DE3) and recoded TAG-less strain MCJ.1217 (C321.ΔA.mutS^{+}.Δλred.Δupp). Error bars = 1 SD.

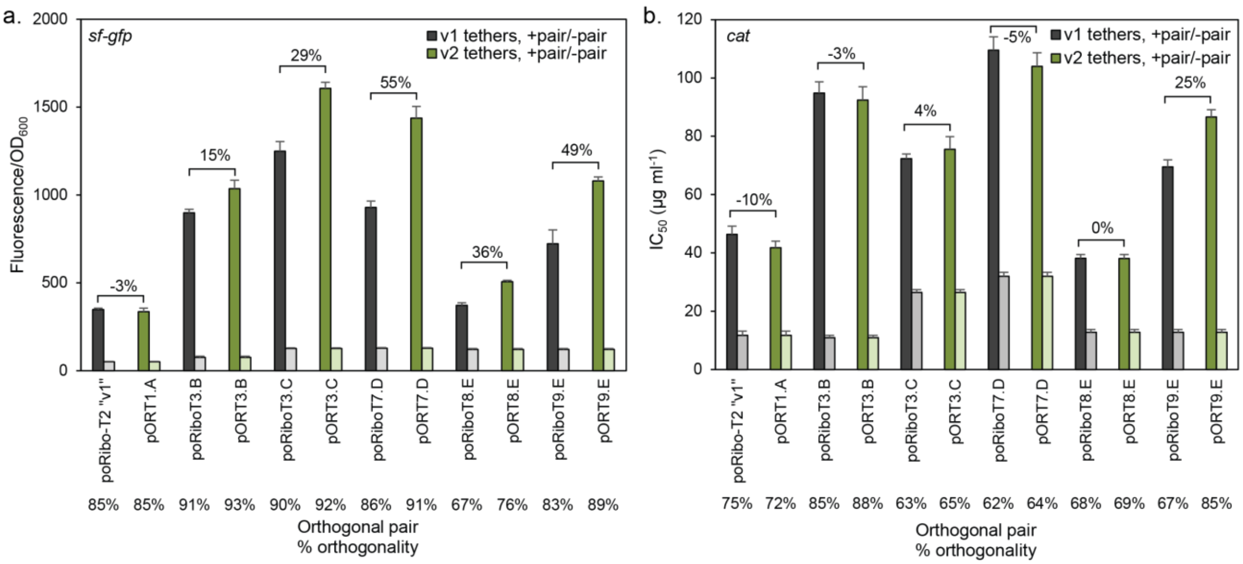

Supplementary Figure 12. Comparison between original published tether (v1) and improved v2 tether with a) *sf-gfp* and b) *cat* reporters. poRibo-T2 is the original orthogonal Ribo-T as published. poRiboT*x.y* contain v1 tethers and pORT*x.y* contain v2 tethers, where *x* indicates rRNA anti-Shine Dalgarno (ASD) species and *y* indicates the cognate mRNA SD species. The bars with corresponding percent values above each set of data represents the percent improvement of the orthogonal pairs with v2 tethers vs. v1 tethers. Error bars = 1 SD.

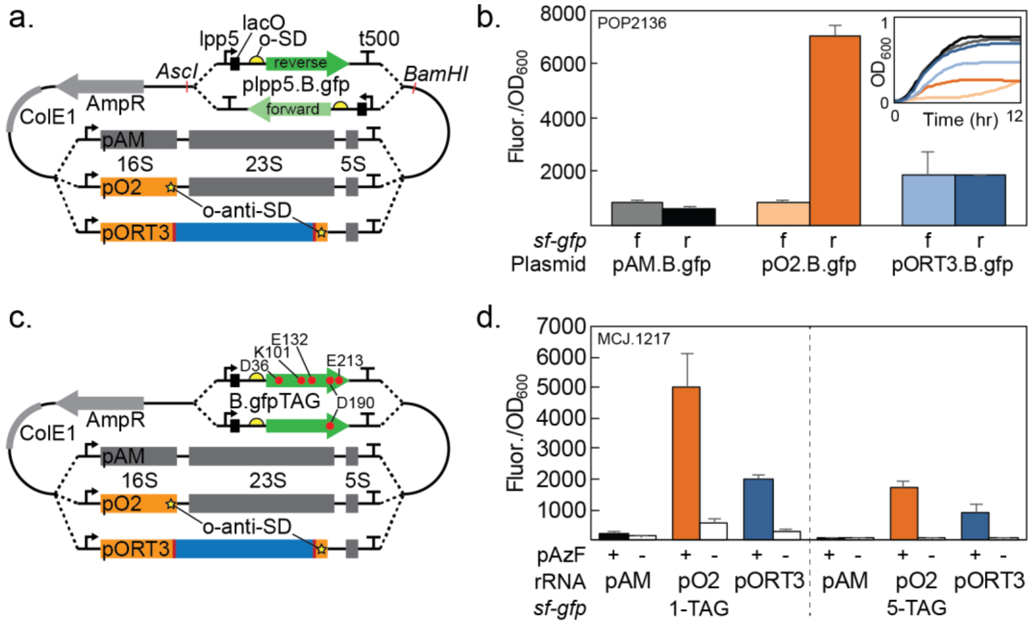

Supplementary Figure 13. Incorporation of non-canonical amino acid p-azidophenylalanine (pAzF) by orthogonal ribosomes. a. Combined rRNA and sfGFP reporter plasmid system. The orthogonal reporter sfGFP is inserted in the forward (light green) and reverse (dark green) direction relative to the *rrn* operon. b. Expression of orthogonal *sf-gfp* from a combined plasmid in POP2136. Representative ribosome constructs include pAM = wild-type untethered ribosomes, pO2 = orthogonal untethered ribosomes possessing optimized ASD sequence 2 (3'-UGGUGU), and pORT3 = orthogonal Ribo-T possessing optimized ASD sequence 3 (3'-GGUGUC). Each construct was combined with the orthogonal sfGFP reporter B (5'-CAACCAC) in the forward (f) or reverse (r) orientation. Fluorescence is normalized by OD_{600} and is the average of at least 3 independent colonies. Error bars = 1 SD. c. Combined rRNA and *sf-gfp* plasmid with reverse orientation. For amber suppression, the *sf-gfp* gene is replaced with a 1TAG or 5TAG version as noted. d. Expression of *sf-gfp* with 1TAG or 5TAG in C321.ΔA.mutS$^+$.Δλred.Δupp (MCJ.1217), in the presence of pAzF (+) or absence of pAzF (-). Fluorescence is normalized by OD_{600} and is the average of at least 3 independent colonies. Error bars = 1 SD.

Supplementary Tables

Supplementary Table 1. Primers used for the construction of Ribo-T tether libraries and gBlocks used in cloning steps.

Primer name	Sequence, 5'-3'
RiboTbb-f	GGAGGGCGCTTACCACTTTG
RiboTbb-r	GGTTAAGCTACCTACTTCTTTTG
T1-A7-f	AAGAAGTAGGTAGCTTAACCAAAAAAATGCGTTGAGCTAAC
T1-A8-f	AAGAAGTAGGTAGCTTAACCAAAAAAAATGCGTTGAGCTAAC
T1-A9-f	AAGAAGTAGGTAGCTTAACCAAAAAAAAATGCGTTGAGCTAAC
T1-A10-f	AAGAAGTAGGTAGCTTAACCAAAAAAAAAATGCGTTGAGCTAAC
T1-A11-f	AAGAAGTAGGTAGCTTAACCAAAAAAAAAAATGCGTTGAGCTAAC
T1-A12-f	AAGAAGTAGGTAGCTTAACCAAAAAAAAAAAATGCGTTGAGCTAAC
T1-A13-f	AAGAAGTAGGTAGCTTAACCAAAAAAAAAAAAATGCGTTGAGCTAAC
T1-A14-f	AAGAAGTAGGTAGCTTAACCAAAAAAAAAAAAAATGCGTTGAGCTAAC
T1-A15-f	AAGAAGTAGGTAGCTTAACCAAAAAAAAAAAAAAATGCGTTGAGCTAAC
T1-A16-f	AAGAAGTAGGTAGCTTAACCAAAAAAAAAAAAAAAATGCGTTGAGCTAAC
T1-A17-f	AAGAAGTAGGTAGCTTAACCAAAAAAAAAAAAAAAAATGCGTTGAGCTAAC
T1-A18-f	AAGAAGTAGGTAGCTTAACCAAAAAAAAAAAAAAAAAATGCGTTGAGCTAAC
T1-A19-f	AAGAAGTAGGTAGCTTAACCAAAAAAAAAAAAAAAAAAATGCGTTGAGCTAAC
T1-A20-f	AAGAAGTAGGTAGCTTAACCAAAAAAAAAAAAAAAAAAAATGCGTTGAGCTAAC
T2-T7-r	CAAAGTGGTAAGCGCCCTCCAAAAAAATGCGCTTACACAC
T2-T8-r	CAAAGTGGTAAGCGCCCTCCAAAAAAAATGCGCTTACACAC
T2-T9-r	CAAAGTGGTAAGCGCCCTCCAAAAAAAAATGCGCTTACACAC
T2-T10-r	CAAAGTGGTAAGCGCCCTCCAAAAAAAAAATGCGCTTACACAC
T2-T11-r	CAAAGTGGTAAGCGCCCTCCAAAAAAAAAAATGCGCTTACACAC
T2-T12-r	CAAAGTGGTAAGCGCCCTCCAAAAAAAAAAAATGCGCTTACACAC
T2-T13-r	CAAAGTGGTAAGCGCCCTCCAAAAAAAAAAAAATGCGCTTACACAC
T2-T14-r	CAAAGTGGTAAGCGCCCTCCAAAAAAAAAAAAAATGCGCTTACACAC
T2-T15-r	CAAAGTGGTAAGCGCCCTCCAAAAAAAAAAAAAAATGCGCTTACACAC
T2-T16-r	CAAAGTGGTAAGCGCCCTCCAAAAAAAAAAAAAAAATGCGCTTACACAC
T2-T17-r	CAAAGTGGTAAGCGCCCTCCAAAAAAAAAAAAAAAAATGCGCTTACACAC
T2-T18-r	CAAAGTGGTAAGCGCCCTCCAAAAAAAAAAAAAAAAAATGCGCTTACACAC
T2-T19-r	CAAAGTGGTAAGCGCCCTCCAAAAAAAAAAAAAAAAAAATGCGCTTACACAC
T2-T20-r	CAAAGTGGTAAGCGCCCTCCAAAAAAAAAAAAAAAAAAAATGCGCTTACACAC
T2-A7-r	CAAAGTGGTAAGCGCCCTCCTTTTTTTTGCGCTTACACAC
T2-A8-r	CAAAGTGGTAAGCGCCCTCCTTTTTTTTTGCGCTTACACAC
T2-A9-r	CAAAGTGGTAAGCGCCCTCCTTTTTTTTTTGCGCTTACACAC
T2-A10-r	CAAAGTGGTAAGCGCCCTCCTTTTTTTTTTTGCGCTTACACAC
T2-A11-r	CAAAGTGGTAAGCGCCCTCCTTTTTTTTTTTTGCGCTTACACAC
T2-A12-r	CAAAGTGGTAAGCGCCCTCCTTTTTTTTTTTTTGCGCTTACACAC
T2-A13-r	CAAAGTGGTAAGCGCCCTCCTTTTTTTTTTTTTTGCGCTTACACAC
T2-A14-r	CAAAGTGGTAAGCGCCCTCCTTTTTTTTTTTTTTTGCGCTTACACAC
T2-A15-r	CAAAGTGGTAAGCGCCCTCCTTTTTTTTTTTTTTTTGCGCTTACACAC

T2-A16-r	CAAAGTGGTAAGCGCCCTCCTTTTTTTTTTTTTTTTTGCGCTTACACAC
T2-A17-r	CAAAGTGGTAAGCGCCCTCCTTTTTTTTTTTTTTTTTTGCGCTTACACAC
T2-A18-r	CAAAGTGGTAAGCGCCCTCCTTTTTTTTTTTTTTTTTTTGCGCTTACACAC
T2-A19-r	CAAAGTGGTAAGCGCCCTCCTTTTTTTTTTTTTTTTTTTTGCGCTTACACAC
T2-A20-r	CAAAGTGGTAAGCGCCCTCCTTTTTTTTTTTTTTTTTTTTTGCGCTTACACAC
T1-8N-f	AAGAAGTAGGTAGCTTAACCTTCGNNNNNNNNCGATGCGTTGAGCTAAC
T2-9N-r	CAAAGTGGTAAGCGCCCTCCGNNNNNNNNNTGCGCTTACACAC
T1-15N-f	AAGAAGTAGGTAGCTTAACCNNNNNNNNNNNNNNNTGCGTTGAGCTAAC
T2-10N-r	CAAAGTGGTAAGCGCCCTCCNNNNNNNNNNTGCGCTTACACAC
p15A gBlock	GATGGCCTTTTTGCGTTTCTACAGAGCGTCAGACCCCTTAATAAGATGATCTTCTTG AGATCGTTTTGGTCTGCGCGTAATCTCTTGCTCTGAAAACGAAAAAACCGCCTTGC AGGGCGGTTTTTCGAAGGTTCTCTGAGCTACCAACTCTTTGAACCGAGGTAACTGG CTTGGAGGAGCGCAGTCACCAAAACTTGTCCTTTCAGTTTAGCCTTAACCGGCGCA TGACTTCAAGACTAACTCCTCTAAATCAATTACCAGTGGCTGCTGCCAGTGGTGCTT TTGCATGTCTTTCCGGGTTGGACTCAAGACGATAGTTACCGGATAAGGCGCAGCGG TCGGACTGAACGGGGGGTTCGTGCATACAGTCCAGCTTGGAGCGAACTGCCTACCC GGAACTGAGTGTCAGGCGTGGAATGAGACAAACGCGGCCATAACAGCGGAATGAC ACCGGTAAACCGAAAGGCAGGAACAGGAGAGCGCACGAGGGAGCCGCCAGGGGG AAACGCCTGGTATCTTTATAGTCCTGTCGGGTTTCGCCACCACTGATTTGAGCGTCA GATTTCGTGATGCTTGTCAGGGGGGCGGAGCCTATGGAAAAACGGCTTTGCCGCGG CCCTCTCGGATCTGTATGGTGCACTCTCAG
lpp5-oRBS	GAGACACAACGTGGCTTTCCATCAAAAAAATATTGACAACATAAAAAACTTTGTGT TATACTTGTGGAATTGTGAGCGGATAACAATTCTATATCTGTTATTTTTCACACCA CAGATCTATGGAGAAAAAAATCACTGGATATACCACCGTTG
