# Supplementary material for: Engineered ribosomes with tethered subunits for expanding biological function
Source: Nat Commun. 2019 Sep 2;10:3920. doi: 10.1038/s41467-019-11427-y (PMC6718428; doi:10.1038/s41467-019-11427-y)
Supplement: Supplementary file 2 — Reporting Summary [file 41467_2019_11427_MOESM2_ESM.pdf]

nature research

Corresponding author(s): Michael Jewett

Last updated by author(s): Jul 9, 2019

Reporting Summary

Nature Research wishes to improve the reproducibility of the work that we publish. This form provides structure for consistency and transparency in reporting. For further information on Nature Research policies, see Authors & Referees and the Editorial Policy Checklist.

Statistics

For all statistical analyses, confirm that the following items are present in the figure legend, table legend, main text, or Methods section.

n/a	Confirmed	
☐	☒	The exact sample size (*n*) for each experimental group/condition, given as a discrete number and unit of measurement
☐	☒	A statement on whether measurements were taken from distinct samples or whether the same sample was measured repeatedly
☐	☒	The statistical test(s) used AND whether they are one- or two-sided<br>*Only common tests should be described solely by name; describe more complex techniques in the Methods section.*
☒	☐	A description of all covariates tested
☒	☐	A description of any assumptions or corrections, such as tests of normality and adjustment for multiple comparisons
☐	☒	A full description of the statistical parameters including central tendency (e.g. means) or other basic estimates (e.g. regression coefficient) AND variation (e.g. standard deviation) or associated estimates of uncertainty (e.g. confidence intervals)
☒	☐	For null hypothesis testing, the test statistic (e.g. *F*, *t*, *r*) with confidence intervals, effect sizes, degrees of freedom and *P* value noted<br>*Give P values as exact values whenever suitable.*
☒	☐	For Bayesian analysis, information on the choice of priors and Markov chain Monte Carlo settings
☒	☐	For hierarchical and complex designs, identification of the appropriate level for tests and full reporting of outcomes
☒	☐	Estimates of effect sizes (e.g. Cohen's *d*, Pearson's *r*), indicating how they were calculated

Our web collection on statistics for biologists contains articles on many of the points above.

Software and code

Policy information about availability of computer code

Data collection	All data were collected using stated instruments and associated commercially available software. No in-house algorithms of software were used to collect the data.
Data analysis	All data were analyzed using stated instruments and associated commercially-available softwares. No custom algorithms or softwares were used for analysis.

For manuscripts utilizing custom algorithms or software that are central to the research but not yet described in published literature, software must be made available to editors/reviewers. We strongly encourage code deposition in a community repository (e.g. GitHub). See the Nature Research guidelines for submitting code & software for further information.

Data

Policy information about availability of data

All manuscripts must include a data availability statement. This statement should provide the following information, where applicable:

- Accession codes, unique identifiers, or web links for publicly available datasets
- A list of figures that have associated raw data
- A description of any restrictions on data availability

All data generated or analyzed in this study are included in this published article (and its supplementary files) or are available from the corresponding authors upon reasonable request.

Field-specific reporting

Please select the one below that is the best fit for your research. If you are not sure, read the appropriate sections before making your selection.

☒ Life sciences ☐ Behavioural & social sciences ☐ Ecological, evolutionary & environmental sciences

For a reference copy of the document with all sections, see nature.com/documents/nr-reporting-summary-flat.pdf

Life sciences study design

All studies must disclose on these points even when the disclosure is negative.

Sample size	Quantification of Ribo-T v2 growth rates and growth rate comparisons were obtained from the mean of multiple (n=3-6) biological replicates. All orthogonality assays, including positive and negative controls, were also acquired from multiple (n=3-6) biological replicates. This includes both liquid-based and plate-based assays. The protein expression experiments were performed in vivo in commercially available cells, BL21 DE3 or BL21 DE3*, or strains available through Addgene or upon request. The ncAA incorporation assay data was obtained from the mean of multiple (n=3-6) biological replicates and measurements were taken at the same time at once.
Data exclusions	No data were excluded.
Replication	All in vivo assays, including growth rates and protein expression, orthogonality assays, and ncAA incorporations at replication were successful.
Randomization	We evolved and characterized an improved Ribo-T system in both the orthogonal and non-orthogonal settings. This improved system was leveraged for ncAA incorporation and orthogonal protein expression. Therefore, no randomization was used or necessary. No animals or human participants were used in this study.
Blinding	Blinding was not relevant because all growth rates, orthogonality assays, and ncAA incorporation assays were fully characterized. No animals or human participants were used in this study.

Reporting for specific materials, systems and methods

We require information from authors about some types of materials, experimental systems and methods used in many studies. Here, indicate whether each material, system or method listed is relevant to your study. If you are not sure if a list item applies to your research, read the appropriate section before selecting a response.

Materials & experimental systems

n/a	Involved in the study	
☒	☐	Antibodies
☒	☐	Eukaryotic cell lines
☒	☐	Palaeontology
☒	☐	Animals and other organisms
☒	☐	Human research participants
☒	☐	Clinical data

Methods

n/a	Involved in the study	
☒	☐	ChIP-seq
☒	☐	Flow cytometry
☒	☐	MRI-based neuroimaging
